# Parenthood in US Medical Training Across Specialty Groups: Scoping Review

**DOI:** 10.2196/87284

**Published:** 2026-07-02

**Authors:** Zuleica M Santiago Delgado, Elizabeth M Vaughan, Mary E Jones, Forrest Jones, Dylan Bridwell, Laura R Porterfield

**Affiliations:** 1 Department of Family Medicine and Community Health, The University of Texas Medical Branch at Galveston, 301 University Boulevard, Galveston, TX, 77555-1123, United States, 1 409 772 0626; 2 Department of Internal Medicine, The University of Texas Medical Branch at Galveston, Galveston, TX, United States; 3 Department of Medicine, Baylor College of Medicine, Houston, TX, United States; 4 Family Medicine Residency Program, Waco Family Medicine Institute, Waco, TX, United States; 5 Family Medicine and Obstetrics Fellowship, Medicos Medical Center, Memphis, TN, United States

**Keywords:** parenthood, graduate medical education, residency, fellowship, lactation, pregnancy, parental leave, social ecological model, childcare, surgical training

## Abstract

**Background:**

Residency and fellowship are demanding phases characterized by intense schedules, limited autonomy, sleep deprivation, and hierarchical environments. Training years often coincide with peak reproductive age, presenting trainees with the dilemma of delaying parenthood or managing training and parenthood concurrently.

**Objective:**

This scoping review examines recent literature on factors influencing the experiences of male and female physician trainee parents across various specialties.

**Methods:**

Four databases were searched for studies published from January 2014 through January 2025 examining pregnancy, parenthood, or lactation outcomes among physician trainees and distinguishing between specialties. Eligible studies were screened, data extracted using predefined criteria, and findings thematically coded. Chi-square and Fisher exact tests assessed specialty-related differences for each theme.

**Results:**

The authors identified 15,861 records, removed 6440 duplicates, and excluded 8989 records. Further, 432 full-text papers were reviewed, with 105 papers included in the final analysis: surgical (n=59), medical subspecialty (n=30), primary care (n=11), and multiple specialties (n=5). Surgical and medical subspecialty studies more often identified interpersonal and policy challenges than primary care. Surgical studies highlighted issues such as pregnancy-related bias, lack of support, rigid schedules, and training-parenthood conflict. Primary care studies more often identified conflicts between parenthood and patient care responsibilities (primary care: 3/11, 27% vs surgical: 3/59, 5% and medical subspecialty studies 0/30, 0%, *P*=.01). Negative well-being impacts from training and parenthood were reported more often in primary care (3/11, 27%) and surgical studies (13/59, 22%) than in medical subspecialties (1/30, 3%, *P*=.03).

**Conclusions:**

Trainees face physician parenthood challenges across social ecological levels, with surgical and subspecialty trainees experiencing more systemic barriers and primary care trainees more patient care–related concerns.

## Introduction

Graduate medical education training (residency and fellowship) represents one of the most demanding phases of a medical career, characterized by long work hours, limited autonomy, inflexible schedules, rigorous requirements, sleep deprivation, and hierarchical workplace structures [1,2]. These years often coincide with peak reproductive age, particularly for women [3]. For physicians, who already work 10 hours more per week than the average US worker, the additional responsibilities of parenthood can be difficult to integrate [4-7]. This strain is even greater for trainees, whose weekly work hours may be more than double that of the typical US worker. Consequently, many physician trainees delay or choose against parenthood [8,9].

Decisions about timing and pursuit of parenthood are influenced by a range of factors, including individual concerns (eg, pregnancy complications, postpartum depression, and perinatal recovery), interpersonal dynamics (eg, peer resentment, supervisor bias, and guilt about neglecting family), and institutional or policy-related barriers (eg, limited parental leave and inadequate lactation accommodations) [10,11]. These challenges are shaped by the specialty context. Surgical, primary care, and nonsurgical medical subspecialties (referred to here as “medical subspecialties”) have varying training demands and practice requirements, with length of training ranging from 3‐5 years in primary care and medical subspecialties to 5‐7 years in surgical specialties [12]. Medical students applying for residency report that their parenthood status significantly influences their specialty choice based on how “family-friendly” a specialty is; however, other factors also strongly influence specialty choice [13].

Prior reviews of physician parenthood have not compared experiences across specialty groups and have included limited male perspectives. A deeper understanding of how parenthood-related challenges differ by training environment could inform cultural, institutional, and policy strategies to more effectively support physician parents. Thus, the objective of this scoping review is to map the literature around experiences, challenges, and interactions of parenthood with training for male and female physicians in residency and fellowship as they relate to individual, interpersonal, organizational, and policy-related factors and their variation across specialty groups.

## Methods

### Study Design

This scoping review was conducted using the PRISMA-ScR (Preferred Reporting Items for Systematic Reviews and Meta-Analyses extension for Scoping Reviews) guideline [14,15]. The protocol was registered and can be found at Open Science Framework Registries [16]. Before initiating the review, a preliminary search of PROSPERO and the Cochrane Database of Systematic Reviews was conducted in January 2025 to ensure no duplicate reviews were underway. Detailed key search terms and a sample search strategy, developed in collaboration with a research librarian, are included in Multimedia Appendix 1; the Medline strategy illustrates the conceptual approach applied across databases, with database-specific adaptations to syntax and indexing. Searches were conducted for studies in English published between January 1, 2014, and January 23, 2025, in Ovid Medline, APA PsycInfo, Scopus, and CINAHL. In addition, references of relevant papers were reviewed for additional potential papers to include.

### Inclusion and Exclusion Criteria

This review was structured using the population, intervention/exposure, comparator, outcome, and study design model: participants (US physician residents and fellows), intervention/exposure (parenthood, pregnancy, and lactation), comparison (surgical, primary care, and medical subspecialty), outcomes (factors influencing experiences including individual, interpersonal, organizational, policy, and interactions between training and parenthood), and study design (peer-reviewed primary studies or systematic/scoping reviews) [17]. Additional inclusion criteria were studies conducted in the United States, published January 2014 through January 2025, and having a primary outcome involving parenthood. Studies were excluded if they did not specifically address parenthood, had a nonphysician population, were conducted outside the United States, or combined outcomes of specialty groups without the ability to distinguish the outcomes. Gray literature, opinion pieces, and conference abstracts were excluded to maintain a focus on empirical findings and avoid the influence of opinion or unconfirmed data.

### Social Ecological Model

#### Overview

**Figure 1.**
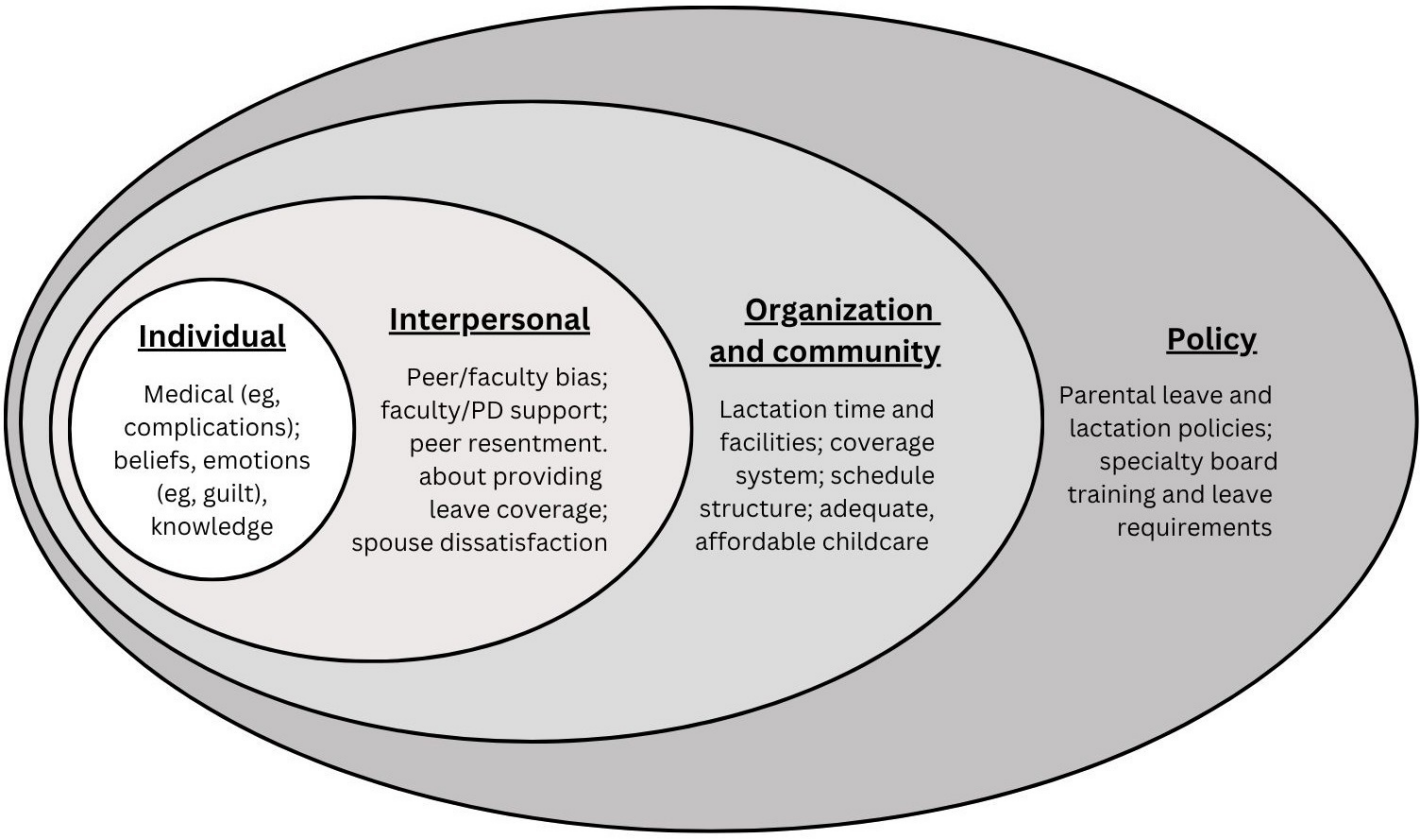
A social ecological model of factors affecting physician parenthood. This figure illustrates a conceptual framework applying the social ecological model to experiences of physician trainee parents. It maps multilevel influences on physician parenthood across four domains: individual, interpersonal, institutional/community, and policy. PD: program director.

The social ecological model (SEM) was used as a framework for understanding the factors affecting decisions about and experiences with physician parenthood. SEM organizes the complex social and environmental factors that shape behaviors and outcomes into levels of influence and explores interactions between levels [18]. For physicians experiencing or considering pregnancy and parenthood, individual factors (eg, self-doubt about ability to cope with both family and work duties and pregnancy complications), interpersonal factors (eg, the perceptions of peers and guilt about peers covering duties during parental leave), community and organizational factors (eg, requirements to make up call shifts that would have taken place during parental leave and availability of adequate childcare services and lactation facilities), and policy factors (eg, parental leave policies) can interact to affect a physician’s experience with pregnancy/parenthood or a physician’s decisions to postpone or avoid parenthood (see Figure 1).

#### Study Selection

Studies were imported into Rayyan, and duplicates were excluded [19]. Two authors independently reviewed the first 500 abstracts and titles for relevance and excluded studies that did not meet the criteria. Interrater reliability for this stage was high (Cohen κ=0.79; 95% CI 0.63‐0.96), with 98.8% agreement. Inclusion of the remaining abstracts was determined by one reviewer. Two authors then independently reviewed the full text of a subset of papers (n=50, 11.6%) for eligibility. Interrater reliability at the full-text stage was lower but remained within the range of substantial agreement (κ=0.61; 95% CI 0.36‐0.86), with 86% agreement. Initial disagreement at the full-text stage most commonly arose in studies with complex or heterogeneous designs requiring interpretive application of prespecified inclusion criteria. These included studies in which outcomes were combined across specialty groups where it required detailed examination of data to determine whether there were separable specialty-specific results (eg, primary care trainees grouped with anesthesiology residents and medical and surgical oncology trainees analyzed together) [20,21]; studies combining United States and international trainee populations where outcomes of interest did not include stratified reporting [22]; studies in which parenthood-related variables were secondary or indirect rather than a primary focus of the study (eg, burnout studies including parental status only as a covariate) [23]; and studies focused on medical students applying to residency (as opposed to residents) [24].

In each discrepant case, reviewers reassessed eligibility using the original prespecified inclusion and exclusion criteria, with particular attention to whether physician trainee parenthood outcomes, specialty-specific analyses, and US-specific data were clearly distinguishable. No post hoc modifications to screening criteria were made during the review process. All discrepancies at both stages were resolved through structured discussion and consensus. The remaining full texts were assessed for eligibility by one author.

#### Data Extraction, Synthesis, and Statistical Calculations

Extracted data were incorporated into an Excel (Microsoft Corp) spreadsheet and included study details (author, year, and title); participant characteristics, demographics, and numbers; studied specialty; and study aim, design, methods, limitations, outcomes, and findings. During data extraction, study results were assigned to a corresponding level of SEM or to an overarching theme of interactions between parenthood and training or career. Data were extracted twice for 15 studies, with the remainder extracted by one author.

A convergent qualitative synthesis approach was used, with SEM serving as a framework to organize and map findings. One author conducted thematic coding to identify recurrent themes, after which themes were reviewed and verified by a second author. To explore specialty-based differences (surgical, primary care, and medical subspecialty), chi-square and/or Fisher exact tests were applied to compare the frequency of each theme by group. For themes in which positive influences on training-parenthood experiences were identified in addition to negative influences, analyses for those themes were repeated while excluding studies with exclusively positive results to highlight differences in areas of challenge across specialty groups.

#### Classification of Specialties

Included studies were categorized into three groups based on specialty type: surgical specialties, medical subspecialties, and primary care. This approach was selected to provide a pragmatic framework that reflects commonly used distinctions in physician workforce and residency experience research [25,26]. We further distinguished medical subspecialties from primary care to preserve clinically meaningful differences in training structure, continuity of care, and patient population. While this classification approach enhances interpretability across heterogeneous studies, it necessarily introduces within-group variability. In particular, some procedural specialties (eg, anesthesiology, interventional cardiology, and interventional gastroenterology) are grouped with primarily cognitive or consultative specialties (eg, psychiatry, neurology, and nephrology), reflecting the absence of a universally accepted classification system in the literature.

To assess the robustness of findings to alternative plausible groupings, we conducted sensitivity analyses reclassifying anesthesiology within the surgical cohort. We compared thematic and statistical outcomes under the original classification scheme (surgical, medical subspecialty, and primary care) with those generated using the alternative classification. This analysis was performed to assess the robustness of findings to plausible alternative specialty groupings and to evaluate whether anesthesiology materially influenced observed between-group differences.

#### Ethical Considerations and Reflexivity

This study was a scoping review of published literature and did not involve human participants or identifiable data. Therefore, institutional review board approval was not required.

The multidisciplinary author team included two medical students without lived experience of parenthood at the time of this study, one family medicine resident father, and three academic faculty (family and internal medicine) who had experienced pregnancy and/or parenthood during residency or fellowship training. Faculty authors also brought experience in residency leadership, core faculty roles, and departmental clinical leadership. These varied perspectives informed the interpretation of findings and encouraged consideration of trainee, parent, educator, and leadership viewpoints throughout the review process.

Although this study did not include a formal stakeholder consultation phase, interpretation of findings was informed through iterative discussions with family medicine program leaders and resident and former resident physician-parents across multiple specialties. Preliminary findings were also presented at the Society of Teachers of Family Medicine Annual Meeting, where feedback from educators and clinicians informed refinement of this paper and interpretation of results.

## Results

### Study Selection and Characteristics

**Figure 2.**
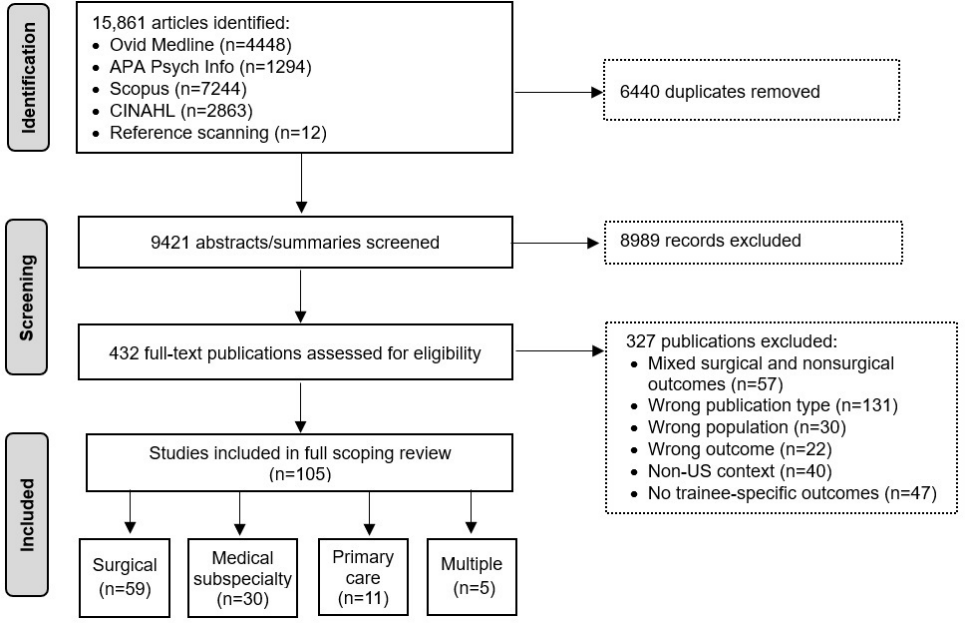
PRISMA flow diagram of paper inclusion and exclusion process summarizes the screening and selection process for the scoping review on physician parenthood. APA: American Psychological Association; CINAHL: Cumulative Index to Nursing and Allied Health Literature; PRISMA: Preferred Reporting Items for Systematic Reviews and Meta-Analyses.

A total of 15,861 papers were identified. After the elimination of 6440 duplicates, 9421 abstracts and titles were assessed for eligibility. Of these, 8989 abstracts did not meet the inclusion criteria, and 432 advanced to the full-text review phase. Of these, 105 were included, with the remaining 327 excluded for the following reasons: wrong population (n=77), lack of a parenthood-related outcome (n=22), non-US context (n=40), lack of separate specialty outcomes (n=57), and wrong publication type (eg, opinion piece; n=131). Included studies were further subdivided by surgical (n=59), medical subspecialty (n=30), primary care (n=11), or multiple specialty groups (n=5). The PRISMA (Preferred Reporting Items for Systematic Reviews and Meta-Analyses) flowchart is included in Figure 2.

The specialties represented in each specialty group are detailed in Table 1. Participants of included studies were predominantly trainees (n=37,986), with former trainees (n=3622) and program directors (PDs; n=3025) also represented. Trainee studies generally included both males and females (n=41 studies), but 16 studies included only females, and 2 only males. Most studies (99/105, 88%) used quantitative methods, particularly surveys (77/105, 73% of studies). Qualitative methods were used in 10/105 (10%) studies, and mixed methods in 3/105 (3%) studies. Primary parenthood-related outcomes included parental leave (38/105, 36% studies); program or colleague support of trainee parenthood or pregnancy (18/105, 17%); health or fertility outcomes (6/105, 6%); trainee parent performance (6/105, 6%); and lactation (4/105, 4%). Almost one-third of studies (33/105, 31%) explored parenthood or pregnancy outcomes across multiple domains.

**Table 1.** Characteristics of included studies.

	All studies	Surgical^a^	Primary care^b^	Medical specialty^c^	Multiple^d^
Number of participants (number of studies)	43,521 (105)	25,529 (59)	2669 (11)	15,948 (30)	375 (5)
Mean (SD)	414 (1279)	416 (1000)	242 (309)	532 (1912)	N/A^e^
Median (IQR)	153 (56-283)	163 (64.5-238.5)	64 (21.5-295.5)	121 (79-205)	N/A
Range	6‐10,803	15‐5692	6‐1021	23‐10,803	N/A
Trainees (studies)	37,986 (59)	22,417 (34)	1781 (8)	13,510 (17)	278 (1)
Female only	16	12	1	3	0
Male only	2	2	0	0	0
Female trainee range (%)	0‐100	0‐100	50‐100	14‐100	64
Graduates (studies)	3622 (18)	2441 (11)	N/A	1181 (7)	N/A
PD^f^ (studies)	3025 (33)	1230 (15)	888 (4)	907 (13)	N/A
Website or policy (studies)	1530 (12)	1084 (6)	N/A	349 (2)	97 (4)
Review studies (studies)	44 (2)	44 (2)	N/A	N/A	N/A
Study methods					
Qualitative, n (%)	10/105 (10)	7	3	N/A	N/A
Focus group	3	2	1	N/A	N/A
Interview	8	5	3	N/A	N/A
Quantitative, n (%)	92/105 (88)	50	7	30	5
Survey	77	46	246	1	1
Quasi-experimental	4	N/A	2	2	N/A
Website, policy, etc	14	6	N/A	4	4
Review	2	2	N/A	N/A	N/A
Mixed, n (%)	3/105 (3)	2	1	N/A	N/A
Parenthood-related outcomes, n (%)					
Parental leave	38/105 (36)	20/59 (34)	2/11 (18)	13/30 (43)	N/A
Trainee support	18/105 (17)	9/59 (17)	2/11 (18)	6/30 (20)	N/A
Health/fertility	6/105 (6)	5/59 (8)	0/11	1/30 (3)	N/A
Performance	6/105 (6)	9/59 (15)	2/11 (18)	13/30 (43)	N/A
Lactation	4/105 (4)	1/59 (2)	1/11 (9)	1/30 (3)	N/A
Multiple	33/105 (31)	18/59 (31)	6/11 (55)	8/30 (27)	N/A

a Surgical (n=59): general surgery (n=18), gynecologic oncology (n=1), multiple (n=13), neurosurgery (n=4), obstetrics and gynecology (n=4), ophthalmology (n=4), orthopedic surgery (n=7), oral & maxillofacial (n=1), otolaryngology (n=1), plastic surgery (n=3), urology (n=2), and vascular surgery (n=1).

b Primary care (n=11): pediatrics (n=7), family medicine (n=3), and internal medicine (n=1).

c Medical subspecialty (n=30): anesthesiology (n=5), cardiology (n=3), dermatology (n=2), emergency medicine (n=2), gastroenterology (n=2), infectious disease (n=1), nephrology (n=1), neurology (n=2), oncology (n=1), physical medicine & rehabilitation (n=1), psychiatry (n=2), radiation oncology (n=4), and radiology (n=4).

d Multiple specialty groups (n=5).

e N/A: not applicable.

f PD: program director.

Details of each study’s population, number of participants, percent female trainees, study design, methodology, and primary outcomes studied are included in Multimedia Appendix 2 [11,21,27-129].

### Interventions

Five studies examined the effect of an intervention for trainee parents using pre- and postintervention data without a control group (Table 2). Interventions included schedule modifications, parental leave policies, parenthood electives, and lactation support. One study of emergency medicine residents instituted schedule changes, including the removal of night shifts for a pregnant resident and flexible scheduling for three expectant fathers, and found no notable negative impact on peer resident schedules [27]. Two studies evaluated the implementation of parental leave policies. An updated parental leave policy in a neurology residency program was associated with higher perceived leadership support of parental leave [28], while a comprehensive parental leave policy in a general surgery program improved perceptions of support and equity surrounding parenthood and training [29]. In a pediatrics residency program, implementation of an at-home neonatal care elective increased the minimum time taken by both child-bearing and nonchildbearing residents without affecting on-time graduation rates [30]. Another pediatrics study installed a hospital-grade pump in an equipped lactation room and reported improved resident engagement in patient care, increased comfort, and decreased guilt and anxiety among lactating residents [31].

Table 3 includes a summary of themes organized by specialty group and SEM level.

**Table 2.** Studies of interventions for trainee parents.

Study	Population (n)	Intervention	Results
Chernoby et al (2021) [27]	Emergency medicine residents (n=79)	Flexible scheduling policy (pregnant and new parent trainees, n=4 in intervention)	86% resident support for pregnancy and 91% for postpartum changes. High satisfaction from the 4 participating residents.
Conway et al (2022) [28]	Neurology residents (n=221)	Revision of parental leave policy: 12-week paid; no extension	Significant increase in perceived policy transparency, consistency, and program leadership support of parental leave.
Corbisiero et al (2024) [29]	General surgery residents (n=65)	Revision of parental leave policy, process, schedules	Significant increase in perception of policy meeting trainee needs, supporting postpartum self-care, policy fairness, and support of childbearing during residency.
Cree-Green et al (2020) [30]	Pediatrics residents (n=64)	At-home neonatal care elective	Increase in minimum time taken by childbearing residents (2 to 6 wk). A total of 79% of nonchildbearing residents took 4+ weeks compared to 0% preintervention. Decrease in unpaid leave.
Creo et al (2018) [31]	Pediatrics (n=6)	Improved lactation room with a hospital-grade pump	Pumping time decreased 8.5 min (95% CI 3.8‐12.2, *P*=.45). Pumping volume increased 2.8 oz (95% CI 1.2‐4.3). Increased patient care engagement, task completion, comfort; decreased anxiety/guilt.

**Table 3.** Summary of themes of included studies.

	Total (N=105), n (%)	Surgical (N=59), n (%)	PC^a^ (N=11), n (%)	MS^b^ (N=30), n (%)	All (N=5)	Values		Fisher exact test (*P* value)
						Chi-square (*df*)	*P* value	
Individual	4946 (46)	31 (53)	3 (27)	15 (50)	0	2.39 (2)	.30	.34
Knowledge/belief	20 (19)	14 (24)	0	6 (20)	0	3.26 (2)	.20	.18
Health	26 (25)	16 (27)	2 (18)	8 (27)	0	0.39 (2)	.82	.89
Fertility	18 (17)	11 (19)	0	7 (23)	0	3.01 (2)	.22	.22
Financial constraints	4 (4)	2 (3)	1 (9)	1 (3)	0	N/A^c^	N/A	.56
Interpersonal	68 (65)	42 (71)	6 (55)	18 (60)	2 (40)	1.83 (2)	.4	.39
Positive n (%)	12 (11)	5 (8)	3 (27)	3 (10)	0	N/A	N/A	N/A
Positive only excluded	62 (59)	41 (69)	3 (27)	16 (53)	0	7.68 (2)^d^	.02	.02^d^
Colleague burden	45 (43)	30 (51)	5 (45)	8 (27)	2 (40)	4.77 (2)	.09	.09
Positive only excluded	44 (42)	30 (51)	4 (36)	8 (27)	2 (40)	4.93 (2)	.09	.09
Bias/stigma	37 (35)	26 (43)	2 (18)	7 (23)	2 (40)	5.29 (2)	.07	.07
Positive only excluded	36 (34)	26 (43)	1 (9)	7 (23)	2 (40)	7.23 (2)^d^	.03	.03^d^
Faculty support	37 (35)	24 (41)	4 (36)	7 (23)17	2 (40)	2.64 (2)	.27	.26
Positive only excluded	32 (30)	24 (41)	1 (9)	5 (17)	2 (40)	8.03 (2)^d^	.02	.02^d^
Peer support	39 (37)	26 (44)	4 (36)	7 (23)	2 (40)	3.67 (2)	.16	.17
Positive only excluded	34 (32)	26 (44)	2 (18)	5 (17)	2 (40)	7.98 (2)^d^	.02^d^	.02^d^
Perceived performance	28 (27)	19 (32)	2 (18)	6 (20)	1 (20)	1.99 (2)	.37	.35
Positive only excluded	25 (24)	19 (32)	1 (9)	4 (13)	1 (20)	5.39 (2)	.07	.09
Patient relationships	7 (7)	3 (5)	4 (36)	0	0	N/A	N/A	.003^d^
Positive only excluded	6 (6)	3 (5)	3 (27)	0	0	N/A	N/A	.01^d^
Family relationships	10 (10)	6 (10)	3 (27)	1 (3)	0	N/A	N/A	.08
Positive only excluded	9 (9)	6 (10)	2 (18)	1 (3)	0	N/A	N/A	.28
Role model/mentors	10 (10)	7 (12)	2 (18)	1 (3)	0	N/A	N/A	.20
Positive only excluded	9 (9)	6 (10)	2 (18)	1 (3)	0	N/A	N/A	.28
Joy of parenthood	2 (2)	0	2 (18)	0	0	N/A	N/A	.01^d^
Organization/community	87 (83)	48 (81)	11 (100)	26 (87)	2 (40)	N/A	N/A	.36
Positive n (%)	20 (19)	5 (8)	8 (73)	7 (23)	0	N/A	N/A	N/A
Positive only excluded	73 (70)	46 (78)	5 (45)	20 (67)	2 (40)	5.15 (2)	.08	.09
Childcare	26 (25)	16 (27)	4 (36)	6 (20)	0	1.21 (2)	.55	.56
Culture of support for pregnancy, parenthood	40 (38)	27 (46)	4 (36)	7 (23)	2 (40)	4.26 (2)	.11	.12
Positive only excluded	38 (36)	27 (46)	2 (18)	7 (23)	2 (40)	6.05 (2)^d^	.049	.05^d^
Culture of support PL^e^	55 (52)	31 (53)	7 (64)	16 (53)	1 (20)	0.47 (2)	.79	.80
Positive only excluded	46 (44)	30 (51)	3 (27)	14 (46)	1 (20)	2.07 (2)	.36	.36
Financial barriers	7 (7)	5 (8)	0	2 (7)	0	N/A	N/A	.86
PL coverage system	37 (35)	17 (29)	5 (45)	15 (50)	0	4.21 (2)	.12	.13
Lactation space	22 (21)	10 (17)	5 (45)	7 (23)	0	4.43 (2)	.11	.11
Positive only excluded	19 (18)	10 (17)	2 (18)	7 (23)	0	0.53 (2)	.77	.81
Lactation time	18 (17)	8 (14)	3 (27)	7 (23)	0	2.01 (2)	.37	.35
Schedule rigor or flexibility	42 (40)	25 (42)	4 (36)	12 (40)	1 (20)	1.34 (2)	.51	.54
Positive only excluded	34 (32)	24 (44)	1 (9)	8 (27)	1 (20)^d^	8.09 (2)^d^	.02^d^	.02^d^
PD^f^ knowledge	10 (10)	4 (7)	1 (9)	5 (17)	0	N/A	N/A	.33
Gender of chair or PD	10 (10)	3 (5)	2 (18)	5 (17)	0	N/A	N/A	.10
Policy	83 (79)	47 (80)	6 (55)	26 (87)	4 (80)	5.04 (2)	.08	.09
Positive (study n)	5 (5)	2 (3)	2 (18)	1 (3)	0	N/A	N/A	N/A
Positive only excluded	79 (75)	46 (78)	4 (36)	25 (83)	4 (80)	19.1 (2)^d^	<.001	<.001^d^
PL policy existence	61 (58)	33 (56)	4 (36)	21 (70)	3 (60)	4.37 (2)	.11	.13
Positive only excluded	58 (55)	32 (54)	3 (27)	20 (67)	3 (60)	6.05 (2)^d^	.05	.06
PL length	52 (49)	28 (47)	4 (36)	16 (53)	4 (80)	0.95 (2)	.62	.64
Positive only excluded	50 (48)	28 (47)	3 (27)	15 (50)	4 (80)	1.8 (2)	.41	.41
Childbearing PL	51 (49)	30 (51)	4 (36)	16 (53)	1 (20)	0.97 (2)	.62	.67
Nonchildbearing PL	37 (35)	21 (36)	3 (27)	12 (40)	1 (20)	0.58 (2)	.75	.83
Nontraditional PL	5 (5)	2 (3)	0	3 (10)	0	N/A	N/A	.84
Paid PL	38 (36)	19 (32)	6 (55)	11 (37)	2 (40)	1.61 (2)	.45	.50
Underuse of PL	17 (16)	8 (14)	3 (27)	6 (20)	0	1.51 (2)	.47	.42
Lactation policy	9 (9)	4 (7)	0	4 (13)	1 (20)	N/A	N/A	.46
Board policy	20 (19)	10 (17)	1 (9)	6 (20)	3 (60)	0.68 (2)	.71	.79
Institutional policy	25 (24)	13 (22)	1 (9)	10 (33)	1 (20)	2.9 (2)	.23	.29
Consistency of policies	13 (12)	9 (15)	1 (9)	3 (10)	0	6.89 (2)^d^	.03	.04^d^
Interactions	78 (74)	44 (75)	7 (64)	24 (80)	3 (60)	1.16 (2)	.56	.55
Positive (study n)	13 (12)	5 (8)	4 (36)	3 (10)	1 (20)	N/A	N/A	N/A
Positive only excluded	71 (68)	40 (68)	6 (55)	22 (73)	3 (60)	1.21 (2)	.52	.49
Training extension	37 (35)	17 (29)	4 (36)	13 (43)	3 (60)	0.32 (2)	.85	.87
Parenthood postponement	28 (27)	18 (31)	1 (9)	8 (27)	1 (20)	2.16 (2)	.34	.43
Training and parenthood incompatibility	12 (11)	11 (19)	1 (9)	0	0	N/A	N/A	.02^d^
Training experience	29 (28)	17 (29)	4 (36)	8 (27)	0	0.37 (2)	.83	.85
Positive only excluded	27 (26)	15 (29)	4 (36)	8 (27)	0	0.57 (2)	.75	.76
Specialty choice	16 (15)	8 (14)	1 (9)	6 (20)	1 (20)	N/A	N/A	.70
Career	28 (27)	15 (25)	3 (27)	10 (33)	0	0.93 (2)	.63	.64
Positive only excluded	21 (20)	11 (19)	2 (18)	8 (27)	0	0.83 (2)	.66	.63
Breastfeeding success	14 (13)	6 (10)	3 (27)	5 (17)	0	N/A	N/A	.22
Positive only excluded	13 (12)	6 (10)	2 (18)	5 (17)	0	N/A	N/A	.61
Combination and well-being/stress	18 (17)	13 (22)	3 (27)	2 (7)	0	3.9 (2)	.14	.10
Positive only excluded	17 (16)	13 (22)	3 (27)	1 (3)	0	5.85 (2)^d^	.05	.03^d^

a PC: primary care.

b MS: medical subspecialties.

c N/A: not applicable.

d Statistically significant at *P*<.05.

e PL: parental leave.

f PD: program director.

### Parenthood-Training Interactions

Three-quarters of included studies (78/105, 74%) described interactions in which training and/or parenthood impacted the other in some way [11,21,27,29-103]. The most common was extension of training as a result of parental leave (37/105, 35%) [30,35,36,38,40,42,44,46,47,49,51,53,57,62-68,70,73,74,76,78,79,81,84-87,90,91,95,96,98,99]. This interaction was particularly concerning for residents planning to pursue a fellowship. Both trainees and PDs described pressure to limit parental leave to avoid extending training as a result of some fellowships not accepting off-cycle residents or viewing off-cycle applicants as less desirable [35,40,46,63,91]. Some studies explored the influence of training on lactation goal achievement (14/105, 13%) [27,29,31,37,39,46,47,54,56,76,85,86,94,99].

Many studies also described postponement or avoidance of parenthood as a result of training (28/105, 27%) [21,29,33,34,37,39,47,48,50,51,54,56,58-60,69,72,75,79-81,87,88,94,95,98,101,102]. For instance, 3 studies of orthopedic trainees found many female trainees (range 48.4%‐68.6%) deliberately postponed childbearing until after training completion, most often to avoid a negative perception by male faculty and cotrainees and to avoid a detrimental career impact [75,87,88]. Studies also described parenthood’s impact on quality of training experience (eg, challenges attaining adequate procedural numbers; 29/105, 28%) [11,31,34,38-40,42,44,45,47,49,54,57,60,71,76,79,81-83,86-88,91,92,95,98,99,103], specialty choice (16/105, 15%) [11,34,43,45,58,60,69,74,79,83,85,88,90,91,99,101], and career success (28/105, 26%) [34,38,41,42,44,46-49,54,57,58,60,69,71,76,77,79,81-83,87,88,91,95,99,102,103]. For example, a study of pediatric residents found female residents with children were significantly more likely to feel limited in their postresidency options because of family considerations compared to both female residents without children and male residents with children [82].

A theme of incompatibility of training and parenthood (12/105, 11%) was more common in surgical studies (11/59, 19%) compared to primary care (1/11, 9%) or medical subspecialties (0/30, 0%, *P*=.02) studies. In 1 study, 32% of female surgical trainees reported parenthood was strongly discouraged: “We were basically told that it would be forbidden to have a family” [80]. In 2 other surgical studies, over a third of female respondents who were pregnant during training (39%‐40%) strongly considered leaving residency due to their pregnancy experience, and a similar number (30%‐33%) would discourage female medical students from pursuing a surgical career because of the challenges of motherhood during surgical training [85,88]. The negative impact on well-being of the combination of parenthood and training (18/105, 17%) was more commonly identified in primary care studies (3/11, 27%) and surgical studies (13/59, 22%) compared to medical subspecialty studies (1/30, 3%, *P*=.03, statistically significant) [11,41,45,49,52,59,60,69,83,88,90,100].

While most interactions were negative, some positives emerged, such as 2 studies in which psychiatry PDs and pediatric trainees described heightened clinical and patient care skills in trainees with parenthood experience [44,99].

### Individual Factors

Individual factors affecting trainee experiences of pregnancy and parenthood were identified in 46% (49/105) of studies [11,21,27,29,31-35,37,39,41,43,45-49,52,54,56,58-60,62,64,68-70,75,76,78,81,85,87,88,90,94,95,97-99,101,102,104-108]. Common themes included health concerns (n=26) such as miscarriage, pregnancy, postpartum, and neonatal complications [11,31,35,37,39,43,46-48,52,54,56,60,64,69,76,78,81,85,88,94,95,97,101,106,107]. For instance, in 1 study, almost two-thirds (63.6%) of general surgery trainees with pregnancies during training believed that their work schedules had harmed their own and/or their child’s health [85]. In another, a majority of female orthopedic residents (60%) cited an inability to ensure optimal prenatal and postnatal care for herself and her child as a barrier to parenthood during residency [88]. Concerns about infertility (n=18) were found in surgical (n=11) and medical subspecialty studies (n=7) but not in primary care studies [21,32-35,37,39,43,58,60,64,81,90,94,95,102,105,108]. Factors involving trainee knowledge (eg, awareness of institutional parental leave policies and knowledge about pregnancy risks of radiation exposure) and beliefs (eg, trainee struggles should be hidden from peers and faculty) were present in 20 studies [21,27,29,33,41,45,46,49,54,56,59,62,68,70,75,87,98,102,104,105]. No statistically significant relationship was found between themes and specialty groups on an individual level.

### Interpersonal Factors

Approximately two-thirds of studies (68/105, 65%) identified interpersonal factors influencing trainee parenthood experiences [11,21,28,31,33-37,39-42,44-49,54-56,58,60-65,67,69,70,72-76,78-81,83,85-90,92,94,95,97-101,103-105,107-115]. When positive interpersonal studies were excluded, surgical (41/59, 69%) and medical subspecialty (16/30, 53%) studies were significantly more likely to identify interpersonal challenges than primary care (3/11, 27%; *P*=.02). The most frequently identified interpersonal theme was the extra burden on peers, who are often expected to cover duties for trainee parents on parental leave (45/105, 43%) [31,33,35,39-42,45-49,54-56,60,62,67,69,70,72,73,75,76,78-81,86,87,89,90,97-99,103-105,107,109-113,115]. In qualitative descriptions, this burden often took the form of peers assuming additional unpaid call or clinical duties to cover parental leave, which trainees described as fostering guilt among trainee parents, and in some cases, resentment or strained relationships among coresidents. Several studies noted that these dynamics discouraged trainees from taking the full parental leave available to them.

Experiences of bias toward trainee parents and experience of being stigmatized because of pregnancy were also common (37/105, 35%), particularly in surgical studies, in which 43% (26/59) identified negative experiences, compared to only 9% (1/11) of primary care studies and 23% (7/30) of medical subspecialty studies (*P*=.03) [11,21,31,33-37,39-41,46,49,54,55,58,60,62,64,69,70,72,74-76,81,83,85,87,88,90,94,95,98,99,103,109]. Reported manifestations included negative or disparaging comments about pregnant trainees and assumptions of reduced skill or commitment. For example, in 1 study, 72.9% of female surgery residents reported witnessing negative comments about pregnant colleagues, and over half perceived a negative stigma associated with pregnancy during training [85].

Other themes included the level of support from a trainee’s faculty (37/105, 35%) [28,31,33,35-37,40,41,44-46,49,54,60,62,69,72,74-76,79-81,83,85-87,92,94,95,99,100,103,104,108,109,112] and fellow trainees (39/105, 37%) [28,31,33,35-37,39-42,44-46,49,55,62,69,70,72,74-76,79-81,83,85-88,94,95,99,100,103,104,108,109,112]. Excluding positive studies, surgical studies were significantly more likely to report issues for these themes (see Table 3).

Additional themes included others’ perceptions about trainee parent performance as a result of their parenthood or pregnancy (28/105, 27%) [11,21,31,34,39-41,44,45,49,54,55,63,65,69,70,72,73,75,85,87-90,98,103,107,113]; the impact of relationships with family members (either support during residency, or strain on the relationship from partners having to shoulder extra home responsibilities; 10/105, 10%) [41,45,61,81,90,99-101,112,114]; the importance of experienced role models and mentors able to help navigate parenthood during training (10/105, 10%) [33,35,41,69,83,85,88,99,108,112]; and, uniquely in primary care studies, the joy of parenthood (2/105, 2%) [99,112]. Primary care studies (3/11, 27%) were significantly more likely than surgical (3/59, 5%) and medical subspecialty (0/30, 0%) studies to highlight challenges around patient relationships as a result of parenthood (*P*=.01) [41,45,61,81,90,99-101,112,114]. For instance, in 1 study, family medicine residents described guilt about having to choose between “going the extra mile” for their patients and leaving for home in time to see their children [112]. However, the number of primary care studies was relatively small (n=11), limiting confidence in specialty-based comparisons and raising the possibility that observed differences may partly reflect sampling variation or publication trends.

### Organizational Factors

The organizational level was the SEM level with the most identified factors influencing trainee parenthood (87/105, 83%) [11,21,27-37,39-42,44-56,58-65,68-70,72-85,87-91,94,95,97-100,102-105,107-123]. Surgical studies were significantly more likely to identify challenges around having a supportive culture for pregnancy and parenthood (27/59, 46% vs 2/11, 18% primary care and 7/30, 23% medical subspecialty, *P*=.05) [11,35,37,39,45-50,52,55,59-61,64,69,72-76,78-81,87-89,94,95,97-100,103,105,107,108,121] and around the rigor and inflexibility of schedules (24/59, 41% vs 1/11, 9% primary care and 8/30, 27% medical subspecialty, *P*=.02) [11,27,33,35,37,39,42,45,47,48,50,53-55,59-61,63,65,68,70,74,76,78,79,81,84,87-90,95,98,100,103,105,107,108,110-112,114,119,122]. For example, surgical program expectations that trainees adhere to demanding call schedules and prolonged work hours up until delivery were noted to be associated with an increased risk of pregnancy complications (eg, preeclampsia and preterm labor) [24].

Themes without significant differences between specialty groups included affordability and adequacy of childcare services and hours (26/105, 25%) [11,32,35,37,45,46,48,58,75,78-80,82-85,87-89,95,99,100,102,108,112,114]; institutional-level financial barriers to supporting paid parental leaves (7/105, 7%) [40,44,48,55,59,65,114]; the system for covering duties of trainees taking parental leave (37/105, 35%) [28,34,41,42,44,46,47,50-55,62-65,68,70,77,79,81,82,90,97,99,103-105,111-115,121-123]; the culture around support for parental leave (55/105, 52%) [21,28-31,33-36,40,41,44-47,49-55,62,64,65,68,69,73-81,84,87-89,94,95,97-99,103-105,109,111,113,120-123]; lactation time (18/105, 17%) [11,35,37,39,47,50,54,56,64,68,75,78,79,89,99,103,108,112], lactation space (22/105, 21%) [31,35,37,39,45,47,50,54,56,64,68,78,79,82-85,88,89,99,108,112]; PD knowledge of board or institutional policies or guidelines (10/105; 10%) [21,51,54,55,65,70,91,99,110,115]; and whether sex of department chair or PD influenced support for trainee parenthood (10/105; 10%) [63,64,73,84,110,111,116-119].

Evidence on how the gender of leadership influences trainee parent experiences was inconsistent across studies. In 1 study, male anesthesiology fellowship directors were more likely to have a negative view of off-cycle applicants [63]. Similarly, 3 studies found that departments with female radiology chairs [116] and residency programs with female anesthesiology [117] or general surgery PDs [84] had higher rates of advertised paid parental leave than those led by males. However, 6 other studies found no significant difference in parental leave support or schedule accommodations based on the gender of department chairs in urology [110], ophthalmology [118], or anesthesiology [64], or of PDs in emergency medicine [111], family medicine [73,119], or anesthesiology [64].

### Policy Factors

Policy factors were the second most common SEM level identified (83/105, 79%) [15,21,28-30,32-37,40-47,49-60,62-68,70,72,75,77-79,81-92,96-99,102,104-111,113,115-119,121-129]. When studies with purely positive findings were excluded, surgical (46/59, 78%) and medical subspecialty (25/30, 83%) studies were significantly more likely than primary care to identify policy-level problems (4/11, 36%, *P*<.001) and were significantly more likely to highlight challenges around the lack of an institutional or program policy (surgical 32/59, 54%, primary care 3/11, 27%; medical subspecialty 20/30, 67%, *P*=.05) [21,28-30,33,34,37,40-46,49,50,52-56,58,59,64,65,67,68,75,79,82,83,85-91,96-99,102,105,107,109-111,113,115-118,121-125,127-129]. Surgical studies were more likely to highlight a lack of alignment of parental leave policies across different levels (eg, specialty board, ACGME [Accreditation Council for Graduate Medical Education], and institutional; surgical 9/59, 15%; primary care 1/11, 9%; medical subspecialty 3/30, 10%; *P*=.03) [33,35,41,43,54,55,87,98,110,113,123]. For instance, the American College of Obstetricians and Gynecologists’ (ACOG) policy recommends at least 8 weeks of paid parental leave in addition to vacation and sick leave [130]. However, in 1 study, 71% of obstetrics and gynecology PDs were unaware of ACOG’s parental leave policy, and local policies were not aligned with ACOG’s policy, resulting in shorter than recommended parental leaves [55]. Similarly, specialty board training requirements may be an obstacle to optimizing institutional policies. For instance, an overwhelming majority (82.2%) of surgery trainees and graduates reported that an American Board of Surgery policy requiring 48 weeks of full-time clinical activity in each of the five years of surgical residency [131] was a barrier to obtaining their desired parental leave length, with over three-quarters (78.4%) receiving a maternity leave of 6 weeks or less [11].

Other policy-related challenges without significant differences between groups included the length of parental leave (52/105, 49%) [28,32-34,36,37,40-42,47,49,51-55,58,60,62,64-68,70,72,77-79,82,85,86,88,89,91,92,96,98,99,102,104,106,108,109,115,118,119,121,122,124-126]; underuse of parental leave (17/105, 16%) [30,34,36,37,40-42,47,51-54,57,65,109,119,122]; and whether parental leave is paid (38/105, 36%) [30,34,40,42,47,52,53,58,62,65,68,70,72,77,78,81,82,84,86,87,96,99,102,104,109,110,113,115-119,121-123,127-129]. Almost half of the studies discussed parental leave for the birthing parent (52/105, 49%) [21,30,34,37,40,42,43,45,47,50-57,59,60,63-66,70,72,75,79,81-89,91,92,98,106,108,110,113,115,116,118,119,121-124,126] and just over a third discussed parental leave for the nonchildbearing parent (37/105, 35%) [34,37,41,42,45,47,51,53-57,62,63,65,66,70,72,82,84,86,87,89,91,98,108-110,113,115,116,118,119,121-124]. Only 5/105 (5%) studies discussed adoptive or nontraditional leave [53,65,121,122,124], and only 9/105 (9%) studies discussed lactation policies [37,53,58,68,86,89,115,121,125]. Institutional-level policies (25/105, 24%) [34,44,46,55,57,58,63-65,72,75,79,85,88,105,110,111,113,115,118,122-124,128,129] were more commonly addressed than specialty board-level policies (20/105, 19%) [33,34,44,46,49,52,54,55,67,82,85,88,91,96,98,110,111,113,125,128].

### Sensitivity Analysis Findings

The sensitivity analysis reclassifying anesthesiology within the surgical specialties demonstrated overall stability in the majority of findings. Differences between specialty groups remained significant for several themes, including bias and stigma (excluding positive-only studies), faculty support, patient relationship, joy of parenthood, and the policy SEM level (excluding positive-only studies).

However, several themes were no longer statistically significant following reclassification. These included the interpersonal SEM level, schedule rigidity, consistency of policy implementation (excluding positive-only studies), perceived compatibility of training and parenthood, and the impact of trainee parenthood on well-being and stress (excluding positive-only studies).

Full results of the sensitivity analysis, including statistical comparisons, are presented in Multimedia Appendix 3.

## Discussion

### Principal Findings

This study applied SEM to map experiences of trainee parenthood across surgical, primary care, and medical subspecialty training. We found that organizational, policy, and interpersonal challenges are more frequently identified in the literature than individual-level barriers. While many challenges were common across specialties, interpersonal and policy-related issues were more prevalent in studies on surgical and medical subspecialty trainees compared to primary care studies. Surgical studies most frequently described stigma surrounding pregnancy and parenthood, a lack of faculty and peer support, rigid schedules, an unsupportive culture of parenthood, and perceived incompatibility between parenthood and training.

The challenges identified in this study align with prior literature, which has consistently described issues such as medical complications of pregnancy, concerns about faculty and peer bias, heavy workloads, rigid schedules, unclear or inadequate parental leave, insufficient lactation facilities, inadequate childcare support, and potential impact on training and career plans [3,8,9,112,132-137]. While no reported prior reviews have compared parenthood experiences across specialty groups, a 2019 systematic review of surgical trainees found that female surgical trainees gave birth to their first child later in life, had fewer children, and were more likely to experience infertility than women in the general population, and were also less likely to have children than male surgical trainees [138]. Although pregnant surgical trainees performed comparably on examinations and case numbers and did not have higher attrition rates, they faced stigma and negative attitudes from colleagues [138]. These results are consistent with our findings that surgical trainees may be more likely than other trainees to experience issues such as stigma, negative attitudes of individuals in the program, and an unsupportive culture for pregnancy and parenthood.

While surgical studies were more likely to report interpersonal and organizational challenges from trainee parenthood, both primary care and surgical studies were more likely than medical subspecialty studies to report a negative impact on well-being from the combination of parenthood and training. Primary care studies were also the most likely to report conflicts between patient care and family life. The included studies did not explicitly explain this finding, which should be interpreted cautiously given the small number of primary care studies. Several hypotheses may warrant future investigation. Relational continuity is a defining feature of primary care, and ongoing therapeutic relationships with patients may increase trainees’ sense of responsibility toward patients when competing family demands arise [139,140]. In addition, primary care work frequently extends beyond scheduled clinical encounters through activities such as inbox management, medication refills, laboratory follow-up, and care coordination [141]. These responsibilities have been associated with after-hours work and erosion of work-life boundaries, which may contribute to work-family conflict [142]. However, it is also possible that differences in study focus, measurement, or reporting practices contributed to this finding rather than true differences between specialty groups.

### Interactions Between SEM Levels

A key strength of the SEM is its ability to illustrate how policy, organizational, interpersonal, and individual factors interact to shape trainee experiences, outcomes, and behaviors. Across the included studies, these levels rarely operated independently; instead, policies were enacted through institutional structures that shaped interpersonal dynamics, which in turn influenced individual decision-making and well-being.

**Figure 3.**
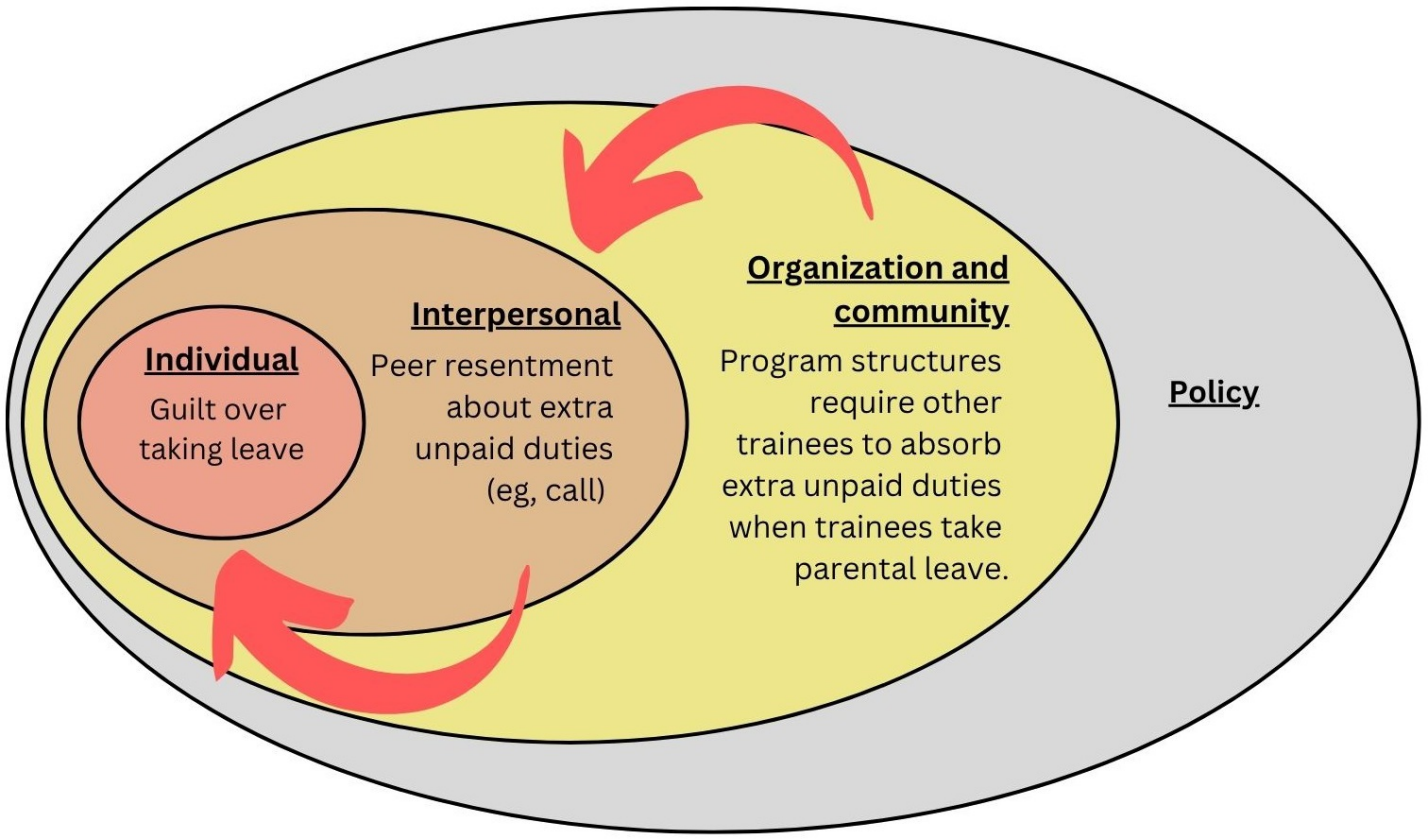
Program structure, interpersonal resentment, and trainee guilt. This figure illustrates how organizational-level factors (eg, training program schedules and coverage systems) interact with interpersonal dynamics (eg, peer resentment), which in turn shape individual-level outcomes such as trainee guilt related to taking parental leave.

For example, parental leave policies and specialty board training requirements at the policy level determine how leave is operationalized within residency programs. Where coverage structures are not formally built into training systems, organizational implementation often shifts the burden of parental leave onto cotrainees, who absorb additional clinical duties without compensation or workload adjustment. This structural arrangement produces predictable interpersonal consequences, including resentment or “colleague burden” among peers covering additional call shifts, and guilt or moral distress among trainee parents taking leave. Over time, these interpersonal dynamics contribute to hesitancy to use available leave, even when policies formally permit it, thereby limiting the practical impact of supportive policy structures (Figure 3). Further, 1 study of female trainees found that even when parental leave was formally available, over half (52.1%) reported not feeling able to take leave, and among those who did, the majority (76%‐88%) reported the leave duration was insufficient [138]. Shortened parental leaves may have downstream consequences; 1 study found that female trainees who took shorter leaves (4‐6 wk instead of 6‐8 wk) were more likely to experience postpartum depression [143].

**Figure 4.**
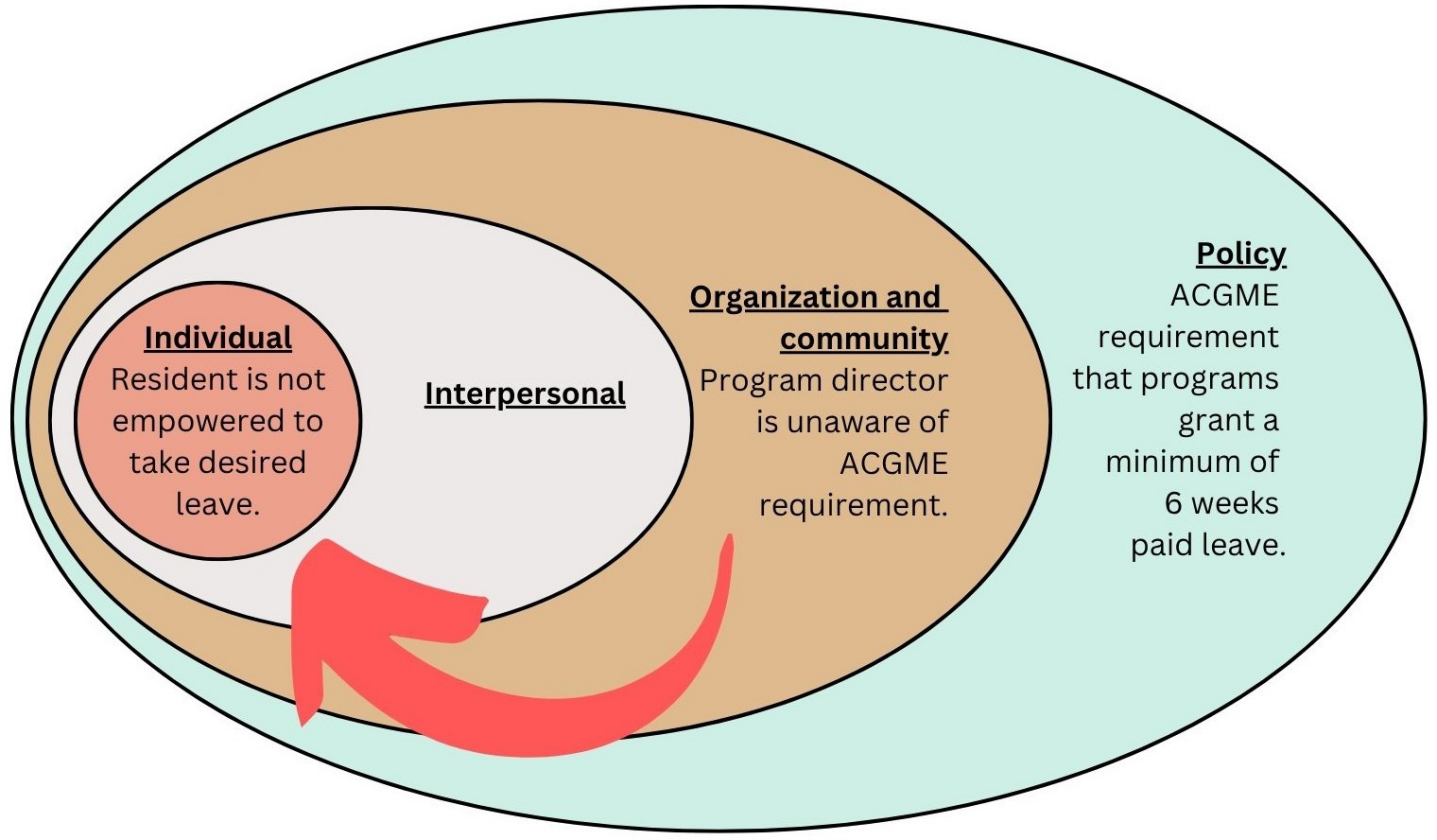
Program director policy awareness. This figure illustrates how, despite the existence of protective parental leave policies, organizational-level barriers (eg, limited program director awareness of these policies) may undermine their implementation and result in negative trainee outcomes, including inability to take desired parental leave. ACGME: Accreditation Council for Graduate Medical Education.

**Figure 5.**
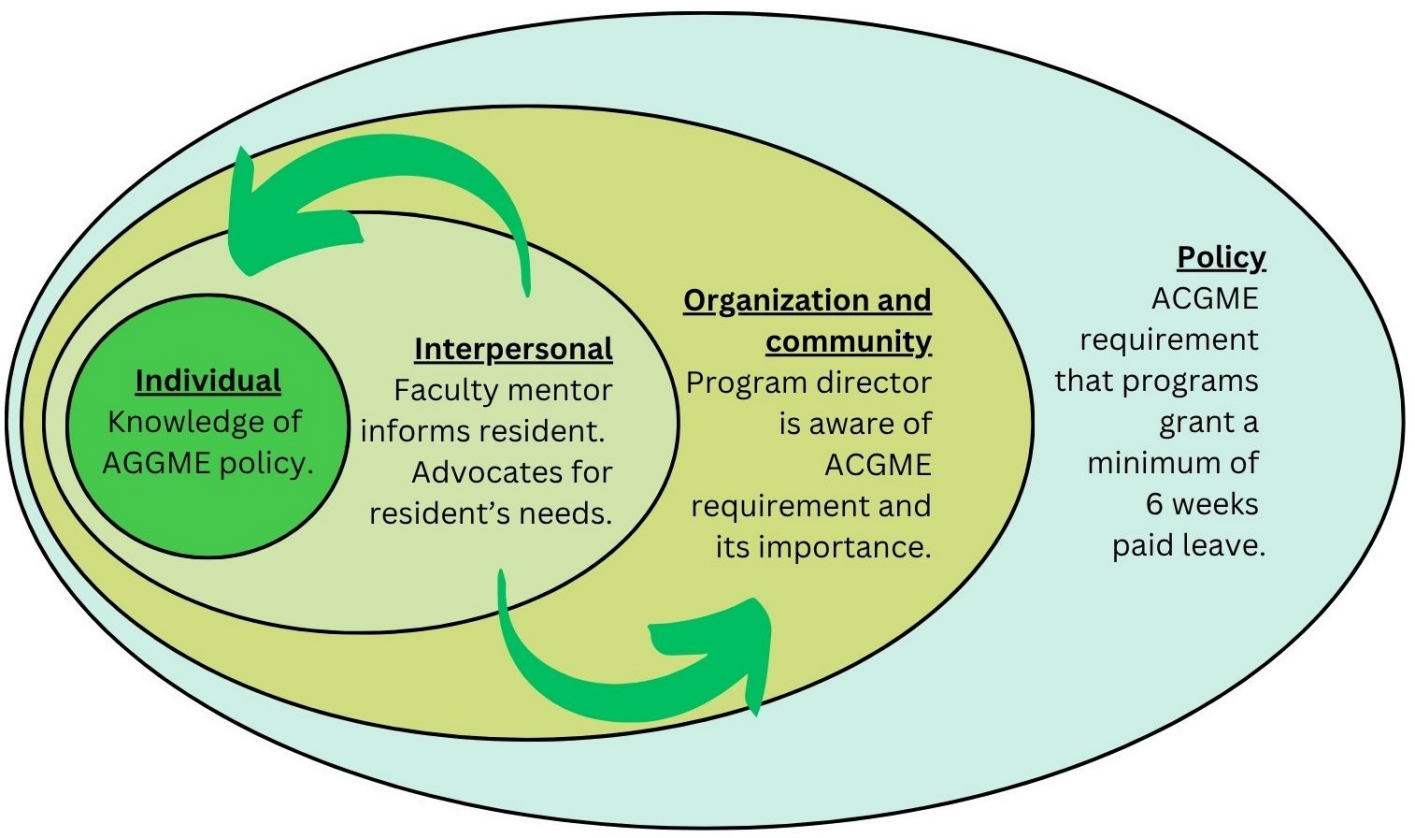
Faculty mentor influence on resident knowledge and organizational support. This figure illustrates how a faculty mentor (an interpersonal-level factor) can function as a positive catalyst for trainee parental leave experiences. Faculty mentors may promote awareness of relevant policies (eg, ACGME requirements for a minimum of 6 weeks of paid parental leave) among both trainees and program leadership, thereby enhancing trainee knowledge and empowerment as well as strengthening organizational support for parental leave. ACGME: Accreditation Council for Graduate Medical Education.

Conversely, these cross-level interactions may also operate in a protective direction. Although many PDs are unaware of ACGME parental leave requirements, limiting consistent implementation (Figure 4), knowledgeable faculty mentors at the interpersonal level may help translate policy into actionable guidance for trainees, reinforce entitlement to leave, and advocate for equitable implementation at the organizational level. In these contexts, supportive interpersonal relationships can reduce stigma and mitigate the effects of organizational ambiguity (Figure 5). However, the literature suggests that this translation function is inconsistent, resulting in variable trainee experiences despite similar formal policies.

Taken together, these findings suggest that misalignment across SEM levels, particularly between policy intent and organizational implementation, may be a key mechanism through which structural conditions produce interpersonal strain and stigma, which in turn shape trainee behavior regarding leave usage and perceived legitimacy of parenthood during training.

### Interventions

Only 5 uncontrolled studies evaluated interventions to improve trainee parenthood experiences. The interventions are clustered into three broad operational approaches. First, several programs modified scheduling and coverage structures. These included removal of overnight call during pregnancy, flexible scheduling for expectant parents, cross-coverage systems, and dedicated parenthood electives that allowed residents to remain engaged in educational activities while spending time at home with a newborn. The available studies suggest that accommodations such as schedule redesign, modified call structures, and parenthood electives may be feasible within existing residency training structures. Notably, the studies that evaluated operational outcomes did not identify adverse effects on resident scheduling or progression, although these outcomes were assessed in only a small number of programs.

Second, programs implemented formal parental leave policies and transparent leave processes. Studies evaluating these interventions reported improvements in perceived leadership support, fairness, and equity surrounding parenthood and training. These findings suggest that clear, standardized parental leave policies may influence trainee perceptions of institutional support, fairness, and equity surrounding parenthood during training.

Third, interventions addressing lactation support focused on reducing logistical barriers to breastfeeding through dedicated spaces and equipment. These interventions were associated with reduced guilt and anxiety and improved ability to remain engaged in clinical responsibilities while meeting lactation needs.

Overall, the limited interventional literature suggests that schedule flexibility, formalized leave policies, mentorship, and lactation infrastructure are the most consistently tested program-level strategies, though evidence remains preliminary.

Our findings underscore the need for multilevel interventions to support trainee parents. Interpersonal dynamics remain critical, especially for females, who are more likely to fear that parenthood will damage their career and professional image and burden peers [34,46-49,54,60,69,70,81,82,85,87,102,103,138]. Organizational and policy factors such as schedule flexibility and paid parental leave also play a vital role. Numerous studies highlight the importance of physician-parent mentorship in balancing family and professional duties [33,35,41,69,83,85,88,99,108,112]. Subsidized childcare can reduce financial burden and stress, especially for female trainees, for whom childcare often emerged as a more significant challenge than for male trainees [10,32,46,48,56,75,79,85,95,114]. Institutions should support lactating trainees through measures such as clear policies, accessible spaces, and schedule accommodations [20,144]. In addition, board policies and training requirements must evolve to protect rather than hinder parenthood during training. For example, if supported by factors at other social ecological levels, ACGME’s 2022 parental leave policy can help ensure trainees are offered at least six weeks of paid leave [145].

We found that 33 of 105 (31.4%) studies reported at least one positive finding related to trainee parenthood experiences, while 103 of 105 (98.1%) studies included at least one negative outcome or challenge. The predominance of studies emphasizing negative outcomes may reflect publication bias, selective outcome framing, or a scholarly tendency to prioritize documenting barriers and strain in trainee parenthood. This imbalance is important because it may shape perceptions of parenthood during medical training. If the published literature disproportionately highlights stress, conflict, and barriers, it may inadvertently reinforce a narrative that parenthood and residency are inherently incompatible. Such framing could influence trainee specialty selection, discourage disclosure of parenting needs, and shape institutional policy discussions toward mitigation of harm rather than optimization of supportive environments. At the same time, the presence of positive findings in nearly one-third of studies suggests that supportive or adaptive experiences are not uncommon, even if they are less frequently reported than challenges.

### Strengths and Limitations

Strengths of this study include the use of a structured framework (SEM), inclusion of male trainee perspectives, and comparison of physician parenthood experiences across specialty groups. However, several limitations reflect gaps in existing literature. Included studies varied widely in design, populations, and quality, with a predominance of survey-based studies increasing the risk of response and selection bias. Notably, studies directly comparing PD and trainee perspectives highlighted marked differences, with PDs reporting substantially more favorable perceptions of program-level support than trainees [21,54]. This finding highlights limitations of stakeholder self-report and raises concerns about measurement validity in the current evidence base.

Only 5 of the included studies evaluated explicit interventions, with the remaining literature largely descriptive, limiting the ability to draw conclusions about effective strategies to support trainee parents. Additionally, exclusion of gray literature may have limited capture of relevant policy-level data, including specialty board guidance and institutional policy statements. Although male trainees were included, the literature remains heavily skewed toward female trainee perspectives, and the positive aspects of parenthood during training were infrequently reported, suggesting possible negativity bias in the existing literature. Some specialties were underrepresented, and within-group heterogeneity (eg, differences between general cardiology and interventional cardiology) may obscure the influence of highly specific training environments on parenthood experiences.

Sensitivity analyses reclassifying anesthesiology within a surgical cohort demonstrated overall stability in the majority of findings, with attenuation of several interpersonal and well-being–related domains. These results suggest that while core thematic differences are robust to alternative classification schemes, certain constructs may be partially sensitive to how procedural specialties are grouped, underscoring the methodological trade-offs inherent in specialty categorization across heterogeneous training environments.

Despite these limitations, this scoping review provides valuable insights into how physicians experience parenthood across individual, interpersonal, organizational, and policy levels during training, and how this may be similar and different across specialty groups.

### Future Research

Future research should prioritize rigorous evaluation of interventions designed to improve trainee parenthood experiences, moving beyond descriptive studies to assess effectiveness. In particular, intervention-focused work should explore which modifiable features of training (eg, specific forms of schedule flexibility, lactation support infrastructure, mentorship models, and childcare resources) are associated with improved trainee well-being, training outcomes, and retention. Incorporating objective institutional metrics will be essential to complement self-reported perceptions and to better understand discrepancies between trainee and program leadership perspectives.

Additional studies are needed to explore subspecialty-specific training environments, including nonsurgical procedural and high-acuity fields such as anesthesiology, where unique occupational exposures and demands may shape parenthood experiences and decisions. Greater inclusion of male trainees and a more balanced examination of both challenges and positive dimensions of parenthood during training would further strengthen the evidence base.

Finally, multi-institutional, international, and longitudinal studies—including posttraining follow-up—may clarify how training-era policies influence long-term physician career trajectories, family decisions, and workforce sustainability [146].

### Conclusions

Trainee parents in the United States face significant individual, interpersonal, organizational, and policy challenges. While many of these are shared across specialties, surgical trainees more frequently report stigma, rigid schedules, and cultural barriers, while primary care trainees often struggle with the tension between patient care and parenting. Effective support for trainee parents requires systemic change. Systemic interventions such as equitable parental leave, childcare support, and cultural shifts in program parenting support are essential to enable all trainees to thrive as both physicians and parents.

## Supplementary material

10.2196/87284 Multimedia Appendix 1 Supplement: search strategies.

10.2196/87284 Multimedia Appendix 2 Summary of individual studies’ characteristics.

10.2196/87284 Multimedia Appendix 3 Sensitivity analysis of specialty-based themes with anesthesiology reclassified as a surgical specialty.

10.2196/87284 Checklist 1 PRISMA-ScR checklist.

## Abbreviations

ACGME: Accreditation Council for Graduate Medical Education
ACOG: American College of Obstetricians and Gynecologists
PD: program director
PRISMA: Preferred Reporting Items for Systematic Reviews and Meta-Analyses
PRISMA-ScR: Preferred Reporting Items for Systematic Reviews and Meta-Analyses extension for Scoping Reviews
SEM: social ecological model
