# Supplementary material for: Parenthood in US Medical Training Across Specialty Groups: Scoping Review
Source: JMIR Med Educ. 2026 Jul 2;12:e87284. doi: 10.2196/87284 (PMC13325462; doi:10.2196/87284)
Supplement: Multimedia Appendix 1 [file mededu-v12-e87284-s001.docx]

**Search Strategy: Ovid Medline**

1. exp Physicians/ or (physician* or doctor*).mp.
2. exp "Internship and Residency"/ or (internship* or resident or residents or residency or residencies or house staff).mp.
3. (allergist* or anesthesiologist* or anesthetist* or anaesthetist* or cardiologist* or dermatologist* or endocrinologist* or gastroenterologist* or "general practitioner*" or geriatrician* or gerontologist* or gynecologist* or gynaecologist* or hepatologist* or hospitalist* or nephrologist* or neurologist* or obstetrician* or oncologist* or ophthalmologist* or orthopedist* or orthopaedist* or otolaryngologist* or otorhinolaryngologist* or otologist* or pathologist* or pediatrician* or paediatrician* or neonatologist* or pulmonologist* or radiologist* or rheumatologist* or surgeon* or neurosurgeon* or urologist*).mp.
4. 1 or 2 or 3
5. exp Parents/ or (parent or parents or parental or parenting or parenthood or mother or mothers or motherhood or father or fathers or fatherhood).mp.
6. exp Pregnancy/ or exp Pregnant women/ or (pregnan* or childbear* or child-bear*).mp.
7. exp Child Rearing/ or (child-rearing or childrearing).mp.
8. exp Breast Feeding/ or exp Lactation/ or exp Milk, Human/ or (breastfeed* or breastfed* or chestfeed* or chestfed* or lactation).mp. or ((breast or chest) adj3 (feed* or fed* or milk)).mp.
9. 5 or 6 or 7 or 8
10. exp Employment/ or (employment or employed).mp.
11. exp Job Satisfaction/ or ((job or work or career) adj2 (satisf* or fulfill* or gratif* or unsatisf* or unfulfill*)).mp.
12. exp Burnout, Professional/ or (burnout or "burn out").mp.
13. exp Occupational Stress/ or ((job or work or career or workplace or occupational or workrelated or "work-related" or professional) adj2 (stress* or abus* or bull*)).mp.
14. (emotional strain* or (feel* adj2 (inadequa* or insuffic* or "not enough"))).mp.
15. exp Workload/ or (workload* or "work load*" or overwork or "over work" or "extra call" or "extra service" or overtime or "service requirement*" or "work hours").mp.
16. exp Career Mobility/ or (((career or job) adj2 mobilit*) or (hiring or firing or hired or fired or tenure* or attrition or quitting or "clinical ladder*" or "career ladder*" or "job ladder*")).mp.
17. exp Workplace/ or (workplace* or "work place*").mp.
18. exp Return to Work/ or ("return to work" or "back to work").mp.
19. exp Parental Leave/ or ((maternity or parental or paternity) adj2 leave).mp.
20. exp Family Leave/ or (family adj2 leave).mp.
21. exp Interprofessional Relations/ or interprofessional relation*.mp.
22. (support adj2 (coworker* or co-worker* or supervisor* or attending*)).mp.
23. exp Social Support/ or social support.mp.
24. exp Social Perception/ or (social adj2 perception*).mp.
25. exp Guilt/ or guilt*.mp.
26. exp Prejudice/ or prejudice*.mp.
27. exp Social Discrimination/ or discriminat*.mp.
28. (promot* and (job* or work* or career* or occupation*)).mp.
29. ((salar* or income* or wage*) adj2 (increase* or decrease* or change*)).mp.
30. earning potential*.mp.
31. ((relative value unit* or rvu or rvus) adj2 (prorate* or decrease* or lower or increase or raise*)).mp.
32. 10 or 11 or 12 or 13 or 14 or 15 or 16 or 17 or 18 or 19 or 20 or 21 or 22 or 23 or 24 or 25 or 26 or 27 or 28 or 29 or 30 or 31
33. 4 and 9 and 32
34. limit 33 to yr="2014 -Current"

**Search Strategy: APA PsycInfo** *(1806 to January 2025 Week 2)*

1. exp Physicians/ or (physician* or doctor*).mp.
2. exp Medical Residency/ or (internship* or resident or residents or residency or residencies or house staff).mp.
3. (allergist* or anesthesiologist* or anesthetist* or anaesthetist* or cardiologist* or dermatologist* or endocrinologist* or gastroenterologist* or "general practitioner*" or geriatrician* or gerontologist* or gynecologist* or gynaecologist* or hepatologist* or hospitalist* or nephrologist* or neurologist* or obstetrician* or oncologist* or ophthalmologist* or orthopedist* or orthopaedist* or otolaryngologist* or otorhinolaryngologist* or otologist* or pathologist* or pediatrician* or paediatrician* or neonatologist* or pulmonologist* or radiologist* or rheumatologist* or surgeon* or neurosurgeon* or urologist*).mp.
4. 1 or 2 or 3
5. exp Parents/ or (parent or parents or parental or parenting or parenthood or mother or mothers or motherhood or father or fathers or fatherhood).mp.
6. exp Pregnancy/ or exp Expectant Mothers/ or (pregnan* or childbear* or child-bear*).mp.
7. (child-rearing or childrearing).mp.
8. exp Breast Feeding/ or exp Lactation/ or (breastfeed* or breastfed* or chestfeed* or chestfed* or lactation).mp. or ((breast or chest) adj3 (feed* or fed* or milk)).mp.
9. 5 or 6 or 7 or 8
10. exp Employment/ or (employment or employed).mp.
11. exp Job Satisfaction/ or ((job or work or career) adj2 (satisf* or fulfill* or gratif* or unsatisf* or unfulfill*)).mp.
12. exp Job Burnout/ or (burnout or "burn out").mp.
13. exp Occupational Stress/ or ((job or work or career or workplace or occupational or workrelated or “work-related” or professional) adj2 (stress* or abus* or bull*)).mp
14. (emotional strain* or (feel* adj2 (inadequa* or insuffic* or "not enough"))).mp.
15. exp Work Load/ or (workload* or "work load*" or overwork or "over work" or "extra call" or "extra service" or overtime or "service requirement*" or "work hours").mp.
16. exp Occupational Mobility/ or (((career or job) adj2 mobilit*) or (hiring or firing or hired or fired or tenure* or attrition or quitting or "clinical ladder*" or "career ladder*" or "job ladder*")).mp.
17. exp Workplace Environment/ or (workplace* or "work place*").mp.
18. exp "Return to Work"/ or ("return to work" or "back to work").mp.
19. ((maternity or parental or paternity) adj2 leave).mp.
20. (family adj2 leave).mp.
21. interprofessional relation*.mp.
22. (support adj2 (coworker* or co-worker* or supervisor* or attending*)).mp.
23. exp Social Support/ or social support.mp.
24. exp Social Perception/ or (social adj2 perception*).mp.
25. exp Guilt/ or guilt*.mp.
26. exp Prejudice/ or prejudice*.mp.
27. exp Social Discrimination/ or discriminat*.mp.
28. (promot* and (job* or work* or career* or occupation*)).mp.
29. ((salar* or income* or wage*) adj2 (increase* or decrease* or change*)).mp.
30. earning potential*.mp.
31. ((relative value unit* or rvu or rvus) adj2 (prorate* or decrease* or lower or increase or raise*)).mp.
32. 10 or 11 or 12 or 13 or 14 or 15 or 16 or 17 or 18 or 19 or 20 or 21 or 22 or 23 or 24 or 25 or 26 or 27 or 28 or 29 or 30 or 31
33. 4 and 9 and 32
34. limit 33 to yr="2014 -Current"

**Search Strategy: Scopus**

( ( INDEXTERMS ( physicians ) OR TITLE-ABS-KEY ( physician* OR doctor* ) ) OR ( INDEXTERMS ( "Internship and Residency" ) OR TITLE-ABS-KEY ( internship* OR resident OR residents OR residency OR residencies OR "house staff" ) ) OR ( TITLE-ABS-KEY ( allergist* OR anesthesiologist* OR anesthetist* OR anaesthetist* OR cardiologist* OR dermatologist* OR endocrinologist* OR gastroenterologist* OR "general practitioner*" OR geriatrician* OR gerontologist* OR gynecologist* OR gynaecologist* OR hepatologist* OR hospitalist* OR nephrologist* OR neurologist* OR obstetrician* OR oncologist* OR ophthalmologist* OR orthopedist* OR orthopaedist* OR otolaryngologist* OR otorhinolaryngologist* OR otologist* OR pathologist* OR pediatrician* OR paediatrician* OR neonatologist* OR pulmonologist* OR radiologist* OR rheumatologist* OR surgeon* OR neurosurgeon* OR urologist* ) ) ) AND ( ( INDEXTERMS ( parents ) OR TITLE-ABS-KEY ( parent OR parents OR parental OR parenting OR parenthood OR mother OR mothers OR motherhood OR father OR fathers OR fatherhood ) ) OR ( INDEXTERMS ( pregnancy ) OR INDEXTERMS ( "Pregnant women" ) OR TITLE-ABS-KEY ( pregnan* OR childbear* OR child-bear* ) ) OR ( INDEXTERMS ( "Child Rearing" ) OR TITLE-ABS-KEY ( child-rearing OR childrearing ) ) OR ( INDEXTERMS ( "Breast Feeding" ) OR INDEXTERMS ( lactation ) OR INDEXTERMS ( "Milk, Human" ) OR TITLE-ABS-KEY ( breastfeed* OR breastfed* OR chestfeed* OR chestfed* OR lactation ) OR TITLE-ABS-KEY ( ( breast OR chest ) W/3 ( feed* OR fed* OR milk ) ) ) ) AND ( ( INDEXTERMS ( employment ) OR TITLE-ABS-KEY ( employment OR employed ) ) OR ( INDEXTERMS ( "Job Satisfaction" ) OR TITLE-ABS-KEY ( ( job OR work OR career ) W/2 ( satisf* OR fulfill* OR gratif* OR unsatisf* OR unfulfill* ) ) ) OR ( INDEXTERMS ( "Burnout, Professional" ) OR TITLE-ABS-KEY ( burnout OR "burn out" ) ) OR ( INDEXTERMS ( "Occupational Stress" ) OR TITLE-ABS-KEY ( ( job OR work OR career OR workplace OR occupational OR workrelated OR work-related OR professional ) W/2 ( stress* OR abus* OR bull* ) ) ) OR ( TITLE-ABS-KEY ( "emotional strain*" OR ( feel* W/2 ( inadequa* OR insuffic* OR "not enough" ) ) ) ) OR ( INDEXTERMS ( workload ) OR TITLE-ABS-KEY ( workload* OR "work load*" OR overwork OR "over work" OR "extra call" OR "extra service" OR overtime OR "service requirement*" OR "work hours" ) ) OR ( INDEXTERMS ( "Career Mobility" ) OR TITLE-ABS-KEY ( ( ( career OR job ) W/2 mobilit* ) OR ( hiring OR firing OR hired OR fired OR tenure* OR attrition OR quitting OR "clinical ladder*" OR "career ladder*" OR "job ladder*" ) ) ) OR ( INDEXTERMS ( workplace ) OR TITLE-ABS-KEY ( workplace* OR "work place*" ) ) OR ( INDEXTERMS ( "Return to Work" ) OR TITLE-ABS-KEY ( "return to work" OR "back to work" ) ) OR ( INDEXTERMS ( "Parental Leave" ) OR TITLE-ABS-KEY ( ( maternity OR parental OR paternity ) W/2 leave ) ) OR ( INDEXTERMS ( "Family Leave" ) OR TITLE-ABS-KEY ( family W/2 leave ) ) OR ( INDEXTERMS ( "Interprofessional Relations" ) OR TITLE-ABS-KEY ( "interprofessional relation*" ) ) OR ( TITLE-ABS-KEY ( support W/2 ( coworker* OR co-worker* OR supervisor* OR attending* ) ) ) OR ( INDEXTERMS ( "Social Support" ) OR TITLE-ABS-KEY ( "social support" ) ) OR ( INDEXTERMS ( "Social Perception" ) OR TITLE-ABS-KEY ( social W/2 perception* ) ) OR ( INDEXTERMS ( guilt ) OR TITLE-ABS-KEY ( guilt* ) ) OR ( INDEXTERMS ( prejudice ) OR TITLE-ABS-KEY ( prejudice* ) ) OR ( INDEXTERMS ( "Social Discrimination" ) OR TITLE-ABS-KEY ( discriminat* ) ) OR ( TITLE-ABS-KEY ( promot* AND ( job* OR work* OR career* OR occupation* ) ) ) OR ( TITLE-ABS-KEY ( ( salar* OR income* OR wage* ) W/2 ( increase* OR decrease* OR change* ) ) ) OR ( TITLE-ABS-KEY ( "earning potential*" ) ) OR ( TITLE-ABS-KEY ( ( "relative value unit*" OR rvu OR rvus ) W/2 ( prorate* OR decrease* OR lower OR increase OR raise* ) ) ) ) AND PUBYEAR > 2013 AND PUBYEAR < 2026

**Search Strategy: CINAHL**

S34 S4 AND S9 AND S32 Limiters - Publication Date: 20140101-20251231

S33 S4 AND S9 AND S32 Search modes

S32 S10 OR S11 OR S12 OR S13 OR S14 OR S15 OR S16 OR S17 OR S18 OR S19 OR S20 OR S21 OR S22 OR S23 OR S24 OR S25 OR S26 OR S27 OR S28 OR S29 OR S30 OR S31

S31 (("relative value unit*" OR rvu OR rvus) N2 (prorate* OR decrease* OR lower OR increase OR raise*))

S30 "earning potential*"

S29 ((salar* OR income* OR wage*) N2 (increase* OR decrease* OR change*))

S28 (promot* AND (job* OR work* OR career* OR occupation*))

S27 (MH "Discrimination+") OR discriminat*

S26 (MH "Prejudice+") OR prejudice*

S25 (MH "Guilt+") OR guilt*

S24 (MH "Social Perception+") OR (social N2 perception*)

S23 (MH "Support, Social+") OR "social support"

S22 (support N2 (coworker* OR co-worker* OR supervisor* OR attending*))

S21 (MH "Interprofessional Relations+") OR "interprofessional relation*"

S20 (MH "Family and Medical Leave+") OR (family N2 leave)

7,702S19 (MH "Parental Leave") OR ((maternity OR parental OR paternity) N2 leave)

S18 (MH "Job Re-Entry") OR ("return to work" OR "back to work")

S17 (MH "Work Environment+") OR (workplace* OR "work place*")

S16 (MH "Career Mobility+") OR (((career OR job) N2 mobilit*) OR (hiring OR firing OR hired

OR fired OR tenure* OR attrition OR quitting OR "clinical ladder*" OR "career ladder*" OR "job ladder*"))

S15 (MH "Workload") OR (workload* OR "work load*" OR overwork OR "over work" OR "extra call" OR "extra service" OR overtime OR "service requirement*" OR "work hours")

S14 ("emotional strain*" OR (feel* N2 (inadequa* OR insuffic* OR "not enough")))

S13 (MH "Stress, Occupational+") OR ((job OR work OR career OR workplace OR occupational OR workrelated OR work-related OR professional) N2 (stress* OR abus* OR bull*))

S12 (MH "Burnout, Professional+") OR (burnout

S11 (MH "Job Satisfaction+") OR ((job OR work OR career) N2 (satisf* OR fulfill* OR gratif* OR unsatisf* OR unfulfill*))

S10 (MH "Employment+") OR (employment OR employed)

S9 S5 OR S6 OR S7 OR S8

S8 (MH "Breast Feeding+") OR (MH "Lactation") OR (MH "Milk, Human+") OR (breastfeed* OR breastfed* OR chestfeed* OR chestfed* OR lactation) OR ((breast OR chest) N3 (feed* OR fed* OR milk))

S7 (MH "Child Rearing+") OR (child-rearing OR childrearing)

S6 (MH "Pregnancy+") OR (MH "Expectant Mothers") OR (pregnan* OR childbear* OR child-bear*)

S5 (MH "Parents+") OR (parent OR parents OR parental OR parenting OR parenthood OR mother OR mothers OR motherhood OR father OR fathers OR fatherhood)

S4 S1 OR S2 OR S3

S3 (allergist* OR anesthesiologist* OR anesthetist* OR anaesthetist* OR cardiologist* OR dermatologist* OR endocrinologist* OR gastroenterologist* OR "general practitioner*" Or geriatrician* OR gerontologist* OR gynecologist* OR gynaecologist* OR hepatologist* OR hospitalist* OR nephrologist* OR neurologist* OR obstetrician* OR oncologist* OR ophthalmologist* OR orthopedist* OR orthopaedist* OR otolaryngologist* OR otorhinolaryngologist* OR otologist* OR pathologist* OR pediatrician* OR paediatrician* OR neonatologist* OR pulmonologist* OR radiologist* OR rheumatologist* OR surgeon* OR neurosurgeon* OR urologist*)

S2 (MH "Internship and Residency+") OR (internship* OR resident OR residents OR residency OR residencies OR "house staff")

S1 (MH "Physicians+") OR (physician* OR doctor*)
