# Supplementary material for: Parenthood in US Medical Training Across Specialty Groups: Scoping Review
Source: JMIR Med Educ. 2026 Jul 2;12:e87284. doi: 10.2196/87284 (PMC13325462; doi:10.2196/87284)
Supplement: Multimedia Appendix 2 [file mededu-v12-e87284-s002.docx]

| Appendix Table: Summary of individual studies’ characteristics | | | | | | | | |
| --- | --- | --- | --- | --- | --- | --- | --- | --- |
|  |  | **Study** | **Study population** | **n** | **% F** | **Design** | **Method** | **Parenthood/pregnancy outcomes studied** |
| Surgical | General surgery | Brown et al [37] 2014 | Categorical general surgery residents | 85 | 42 | DB | Quant | Impact of parenthood on performance and attrition during general surgery residency. |
|  |  | Castillo-Angeles et al [39] 2021 | General surgery PDs | 40 | N/A | I | Qual | Maternity leave and postpartum support for surgical residents (PD perspectives). |
|  |  | Castillo-Angeles [40] 2022a | Male surgical residents | 15 | 0 | I | Qual | Paternity leave during surgical training (male surgical resident perspectives) |
|  |  | Castillo-Angeles [108] 2022b | General surgery PDs | 40 | N/A | I | Qual | Provision of paternity leave in residency (perspectives of surgical program directors) |
|  |  | Corbisiero et al [28] 2024 | General surgery residents | 65 | 55-59 | Survey | Quant | Impact of formal leave policy on trainees' perceptions and wellbeing |
|  |  | Engelbrecht-Wiggans et al [124] 2024 | Gen. surgery websites | 344 | N/A | Website | Quant | Decisions about childbearing, determinants of parental leave, and associations with well-being. |
|  |  | Freudenberger et al [48] 2024 | General surgery faculty | 163 | N/A | Survey | Quant | Training environment for pregnant residents and perceptions of general surgery faculty toward them. |
|  |  | Janczewski et al [60] 2024 | General surgery residents and PDs | 5133 | 44 | Survey, FG/I | Mixed | How programs support work life balance. |
|  |  | Kling et al [61] 2024 | Non-childbearing surgical residents | 20 | 0 | I | Qual | Experiences with paternity leave during clinical years vs research years in surgery residency. |
|  |  | Li et al [59] 2024 | General surgery residents | 5692 | 46 | Survey | Quant | Pregnancy/parenthood experiences, OB outcomes. |
|  |  | Ouyang et al [79] 2021 | Female general surgery grads | 37 | 100 | Survey | Quant | How childbearing experiences have evolved over time for female surgical residency graduates. |
|  |  | Rangel [82] 2018a | Gen surg res who delivered in training | 347 | 100 | Survey | Quant | Factors associated with residency and career dissatisfaction in childbearing surgical residents |
|  |  | Rangel [11] 2018b | Gen surg res who delivered in training | 347 | 100 | Survey | Quant | The experience and perception of women surgeons who have been pregnant during residency. |
|  |  | Rangel et al [84] 2023 | Gen surg programs | 254 | N/A | Website | Quant | The transparency of parental benefits |
|  |  | Sandler et al [88] 2016 | Surgery program directors | 66 | N/A | Survey | Quant | How surgery programs accommodate resident pregnancy and parenthood and PD perspectives. |
|  |  | Shifflette et al [91] 2024 | Gen surg programs | 26 | N/A | Survey | Quant | Pregnancy impact on female residents. |
|  |  | Zhang et al [101] 2024 | Surgical residents | 121 | 79 | Survey | Quant | Surgical trainees’ perspectives on family planning. |
|  |  | Zmijewski et al [102] 2024 | Surgical residents | 22 | 100 | FG | Qual | Impact of parenthood on operative performance for female residents. |
|  | Gyn Onc | Szender et al [92] 2016 | Gyn onc fellows | 167 | 79 | Survey | Quant | Risk factors for dissatisfaction with work life balance, and impact on career plans |
| Surgical | Multiple Surgical Specialties | Gaffley et al [51] 2024 | Trainees in southeast | 164 | 60 | Survey | Quant | Experiences of parental leave, pregnancy timing, and perceived support |
|  |  | Huynh et al [58] 2021 | Residency & fellowship PDs | 299 | N/A | Survey | Quant | Knowledge and support of PDs toward trainees' reproductive needs (e.g., fertility) |
|  |  | Magudia et al [126] 2020 | Trainees from 285 programs | 578 | 61 | Survey | Quant | Trainees parent ability to function as part of a team; amount of PL time taken; satisfaction with PL |
|  |  | Mundschenk et al [73] 2016 | Surgical residents | 277 | 100/51 | Survey | Quant | Workplace culture around childbearing during residency; influence of peers, faculty, & leaders. |
|  |  | Rangel [84] 2018c | Female surgical residents | 347 | 100 | Survey | Qual | Perspectives of pregnant surgical residents; positive influences on experience of childbearing trainees |
|  |  | Tatarian et al [106] 2024 | Surgeons across all surgical specialties | 817 | 100 | Survey | Quant | Association between workload and pregnancy outcomes among US surgical faculty and trainees |
|  |  | Warner et al [96] 2024 | Mixed residents | 967 |  | Survey | Quant | Attitudes toward pregnancy during training; Pregnancy rates and complications. |
|  | Neurosurgery | Parker et al [80] 2021 | Female neurosurgeons | 260 | 100 | Survey | Quant | Perceived career impact of motherhood; timing, complications, and factors affecting pregnancy |
|  |  | Sanusi et al [89] 2024 | Neurosurgery faculty & trainees | 113 | 72 | Survey | Quant | Parental leave in neurosurgery. |
|  |  | Thum et al [93] 2021 | Neurosurgical programs | 115 | 44 | Survey | Quant | Family planning |
|  | Ob-gyn | Esfandiari et al [47] 2019 | OB-gyn residents | 113 | 91 | Survey | Quant | Delays of parenthood, knowledge of oocyte freezing and impact of education. |
|  |  | Hariton et al [54] 2018 | Ob-gyn PDs | 163 | N/A | Survey | Quant | Perspectives about parental leave policies, parenting in residency |
|  |  | Martin et al [105] 2019 | Female ob-gyn faculty and residents | 204 | 100 | Survey | Quant | Reproductive experiences (post-partum, infertility, reproductive planning, birth outcomes). |
|  |  | Mattei et al [69] 2024 | Ob-gyn residents, fellows, and faculty | 454 | 94 | Survey | Quant | Institutional policy and workplace cultural barriers to family building |
|  | Ophthalmology | Huh et al [56] 2022 | Ophthalmology residents | 283 | 47 | Survey | Quant | Performance association with parental leave |
|  |  | Reilly et al [85] 2022 | Ophthalmology residents and PDs | 97 | 42 | Survey | Quant | Resident and PD attitudes toward parental leave; range of parental leave and breastfeeding policies |
|  |  | Tao et al [118] 2024 | Ophthalmology programs | 124 | N/A | Website | Quant | Advertised parental leave benefits |
|  |  | Wang et al [97] 2021 | Ophthalmology PDs | 82 | N/A | Survey | Quant | Parental leave policies and practices |
|  | Orthopedic surgery | Mercurio et al [127] 2024 | Ortho residencies | 170 | N/A | Website | Quant | Parental leave information availability on websites |
|  |  | Mulcahey et al [74] 2019 | Female orthopedic residents | 190 | 100 | Survey | Quant | Perceptions and experiences of female orthopedic trainees with pregnancy and parenthood. |
|  |  | Nguyen et al [76] 2020 | Female ortho surgeons | 801 | 100 | Survey | Quant | Maternity leave characteristics |
|  |  | Reid et al [86] 2021 | Orthopedic residents and PDs | 495 | 24 | Survey | Quant | Family-planning decisions, program policies, and perceptions regarding parenthood in residency |
|  |  | Ruse et al [87] 2022 | Female ortho surgeons | 328 | 100 | Survey | Quant | Barriers to pregnancy during residency |
|  |  | Siljander et al [128] 2020 | Orthopedic programs | 166 | N/A | DB | Quant | Availability of parental leave and provisions |
|  |  | Weiss et al [122] 2016 | Orthopedic PDs | 45 | N/A | Survey | Quant | Parental leave policies; time and makeup options |
|  | OMF | Diaz et al [44] 2021 | Oral & maxillofacial surgery residents | 220 | 29 | Survey | Quant | Formal parental leave, attitudes about colleague parenthood, lactation facilities/childcare services |
|  | ENT | Tang et al [112] 2019 | Otolaryngology PDs | 41 | N/A | Survey | Quant | Existence and nature of parental leave policies for residents during otolaryngology training. |
|  | Plastic | Bourne et al [36] 2019 | Plastic surgery trainees | 307 | 54 | Survey | Quant | Obstetric complications, parental leave, breastfeeding, childcare, infertility. |
|  |  | Garza et al [49] 2017 | Plastic surgery PDs | 54 | N/A | Survey | Quant | Challenges in accommodating pregnant residents |
|  |  | Wallace et al [113] 2022 | Plastic surg residents | 32 | 66 | Survey | Quant | Childrearing during plastic surgery training. |
|  | Multiple Surgical | Acker et al [103] 2024 | Surgery faculty & trainees | 118 | 56 | Survey | Mixed | Parental leave during surgical training |
|  |  | Altieri et al [32] 2019 | Surgical residents | 2188 | 49 | Survey | Quant | Parental leave and pregnancy during residency |
|  |  | Bernal et al [34] 2024 | Surgical residents | 17 | N/A | SR | Qual | Parenthood challenges during surgical training |
|  |  | Bostock et al [35] 2023 | Trainee parents | 64 | N/A | Survey | Quant | Experience of trainee parents |
|  |  | Castillo-Angeles [38] 2022c | Surgical residents and fellows | 258 | 100 | Survey | Quant | Program and colleague support of surgical trainees for pregnancy and postpartum needs. |
|  |  | Mann and Glazer [104] 2024 | Surgical program PDs | 143 | N/A | Survey | Quant | Policies & protections for pregnant surgical trainees |
|  |  | Todd et al [94] 2020 | Surgery programs | 27 | N/A | NR | Quant | Themes and data around pregnancy and parenthood during surgical residency training |
|  | Uro | Kenyon et al [109] 2021 | Urology PDs | 63 | N/A | Survey | Quant | Pregnancy during urology residency |
|  |  | MacDonald et al [114] 2021 | Urology PDs | 65 | N/A | Survey | Quant | Parental leave for urology trainees |
|  | Vasc | Maloni et al [68] 2022 | Vascular surgeons, PDs | 296 | 56 | Survey | Quant | Views of pregnancy in vascular surgeons in training |
| Primary Care | Family Medicine | Morris et al [111] 2016 | Family medicine residents | 8 | 50 | Focus Groups | Qual | Experiences of parenting family medicine residents as parents and physicians |
|  |  | Morris et al [72] 2018 | Family medicine PDs | 298 | N/A | Survey | Quant | Support for trainee parenthood; performance. well-being. |
|  |  | Wendling et al [119] 2019 | Family medicine PDs | 261 | N/A | Survey | Quant | Parental leave policies, actual leave taken, and predicting factors. |
|  | IM | Stack et al [123] 2022 | Internal Medicine PDs | 293 | N/A | Survey | Quant | Parental leave policies, barriers, and PD willingness to implement a national standardized policy |
|  | Pediatrics | Ben-Zion et al [120] 2022 | Pediatric PDs | 36 | N/A | Survey | Quant | Parenting electives for new resident parents |
|  |  | Cree-Green et al [29] 2020 | Pediatric residents | 64 | N/A | Quasi | Quant | Impact of parenting elective on parental leave taken |
|  |  | Creo et al [30] 2018 | Pediatric residents | 6 | 100 | Quasi/I | Mixed | Impact of lactation room and pump on time spent pumping, milk volume, and resident satisfaction |
|  |  | Dundon et al [46] 2021 | Pediatric residents | 639 | 70 | Survey | Quant | Satisfaction with breastfeeding duration, parental leave length, and support |
|  |  | Powell et al [81] 2021 | Pediatric residents | 1021 | 75 | Survey | Quant | Parental benefits and impact on career goals |
|  |  | Wilder et al [98] 2021a | Pediatric residents | 31 | 71 | I | Qual | Experience of parenthood during residency |
|  |  | Wilder et al [99] 2021b | Pediatric residents | 12 | 83 | I | Qual | Parenthood during residency/COVID pandemic |
| Medical Subspecialties | Anesthesiology | Kraus et al [63] 2021 | Anesthesiologists with pregnancy during training | 542 | 100 | Survey | Quant | Childbearing experiences during residency and/or fellowship |
|  |  | Kraus et al [62] 2022 | Anesthesiology fellowship directors | 101 | N/A | Survey | Quant | Perceptions of pregnancy and parental leave during training |
|  |  | McGough et al [107] 2022 | Anes residents | 92 | 44 | Survey | Quant | Attitudes about parental leave during training. |
|  |  | Morah et al [117] 2024 | Anes programs | 164 | N/A | Website | Quant | Leadership gender and paid parental leave |
|  |  | Sharpe et al [90] 2021 | Anesthesiology PDs | 56 | N/A | Survey | Quant | Effect of pregnancy/parental leave on resident training, skills, and productivity. |
|  | Cardiology | Mwakyanjala et al [75] 2019 | Cardiovascular fellows | 29 | 49 | Survey | Quant | Impact of pregnancy, parenthood, and lactation |
|  |  | Oliveros et al [77] 2022 | Cardiovascular trainees and faculty | 193 | 75 | Survey | Quant | Schedules, radiation exposure, parental leave, lactation, childcare, and pregnancy outcomes |
|  |  | Yong et al [100] 2019 | Cardiovascular fellows | 574 | 14 | Survey | Quant | Factors for choosing a career in interventional card |
|  | Derm | Gracey et al [53] 2018 | Derm PDs and residents | 193 | 71 | Survey | Quant | Parental leave policies experiences |
|  |  | Humphrey et al [57] 2024 | Dermatology residents | 95 | 100 | Survey | Quant | Impact of dermatology residency training on family planning, fertility, and career decisions. |
| Medical Subspecialties | EM | Chernoby et al [26] 2021 | Emergency medicine residents | 79 | 25 | Quasi | Quant | Implementation of scheduling process for new parents: satisfaction, and pregnancy outcomes |
|  |  | MacVane et al [110] 2022 | EM PDs and leaders | 167 | N/A | Survey | Quant | Scheduling practices for pregnant EM residents, barriers to instituting a pregnancy scheduling policy |
|  | GI | Advani et al [31] 2025 | IM residents considering GI | 543 | 53 | Survey | Quant | Fertility, maternity/paternity leave, impact of family obligations on subspecialty choice (GI) |
|  |  | David et al [42] 2021 | Gastroenterology fellows and faculty | 332 | 65 | Quasi | Quant | Childcare and issues surrounding childbearing and impact on choice of gastroenterology. |
|  | ID | Gardiner et al [50] 2024 | Infectious disease PDs | 56 | N/A | Survey | Quant | PD interpretation of ABIM leave policies. |
|  | Ne | Dixon et al [45] 2024 | Nephrology fellows | 121 | 62 | Survey | Quant | Parental leave, pregnancy, lactation, childcare accommodations, & family planning |
|  | Neuro | Conway et al [27] 2022 | Neurology residents and former residents | 221 | 44 | Survey | Quant | Effects of a parental leave policy. |
|  |  | Conway et al [41] 2024 | Neurology PDs | 54 | N/A | Survey | Quant | Parental leave policies and barriers to increasing the duration of leave. |
|  | Onc | Malapati et al [67] 2023 | Oncology trainees/PDs | 278 | 54 | Survey | Quant | Experiences of parental leave and lactation policies |
|  | PMR | McDeavitt et al [70] 2022 | Physical medicine and rehabilitation residents | 10803 | 67 | DB | Quant | Leaves (including parental) during PM&R training and exam pass rates |
|  | Psych | Dillinger et al [43] 2022 | Adult psychiatry PDs | 49 | N/A | Survey | Quant | Impact of maternity leave on training |
|  |  | Leandre et al [64] 2022 | Psychiatry PDs | 186 | N/A | Survey | Quant | Parental leave policies and accommodations |
|  | Radiation oncology | Baniel et al [33] 2023 | Radiation oncology PDs & applicants | 91 | 44 | Survey | Quant | Resident parental leave and starting families in residency |
|  |  | Beltran Ponce et al [20] 2022 | Radiation oncology PDs and residents | 80 | 57 | Survey | Quant | Parental leave and parenthood during training |
|  |  | Holliday et al [55] 2015 | Radiation oncology residents graduates | 190 | 44 | Survey | Quant | Parenthood and pregnancy frequency, experiences, and gender disparities. |
|  |  | Osborn et al [78] 2019 | Female radiation oncology residents | 125 | 100 | Survey | Quant | Program support and influence on reproductive plans and parenthood experiences |
|  | Radiology | Ghazi et al [52] 2020 | Radiology PDs | 74 | N/A | Survey | Quant | Parental leave policies, leave lengths, schedule accommodations for pregnant residents |
|  |  | Hui et al [116] 2022 | Radiology chairs | 209 | N/A | Website | Quant | Parental leave policies and gender of chair |
|  |  | Sherbaf et al [121] 2020 | Neuroradiology PDs | 66 | N/A | Survey | Quant | Parental leave policies |
|  |  | Wong et al [130] 2022 | Radiology program websites | 185 | N/A | Website | Quant | Resident wellness initiatives and parental leave |
| Multiple | Multiple | Kasemodel et al [125] 2022 | Specialty websites | 26 | N/A | Website | Quant | Current parental leave and lactation policies of the American Board of Medical Specialties |
|  |  | Lumpkin et al [65] 2019 | Primary board specialty websites | 24 | N/A | Website | Quant | Impact of 6- and 12-week leave on residency training, board eligibility, and fellowship training |
|  |  | Magudia et al [66] 2021 | ABMS boards with 2+ year training pathways. | 23 | N/A | Website | Quant | Publicly accessible board leave policies; time requirements; training extension |
|  |  | Morgan et al [71] 2024 | Trainees (all specialties) | 278 | 64-91 | Survey | Quant | Policy and perceptions of pregnancy during training |
|  |  | Varda and Glover [95] 2018 | Leave policies | 24 | N/A | Website | Quant | Leave policies for residents among ABMS organizations |
| Table Legend | | n: number of study participants  F: % female trainees  PD: program directors  DB: dabase review  I: interviews  FG: focus groups  N/A: not applicable  Quant: quantitative  Qual: qualitative  Quasi: quasiexperimental  Gen surg: general surgery | | Gyn onc: gynecologic oncology  Res: residents  Ob-gyn: obstetrics and gynecology  Ortho: orthopedics  OMF: oromaxillofacial  ENT: otolaryngology  Plastic: plastic surgery  Surg: surgery  Uro: urology  Vasc: vascular surgery  IM: internal medicine | | | | Anes: anesthesiology  Derm: dermatology  EM: emergency medicine  GI: gatroenterology  Neph: nephrology  Neuro: neurology  Onc: oncology  PMR: physical medicine and rehabilitation  Psych: psychiatry  PL: parental leave  ABMS: American Board of Medical Specialties |

### **REFERENCES**

37. Brown EG, Galante JM, Keller BA, Braxton J, Farmer DL. Pregnancy-related attrition in general surgery. JAMA Surg. Sep 1, 2014;149(9):893. [doi: ]

39. Castillo-Angeles M, Smink DS, Rangel EL. Perspectives of US general surgery program directors on cultural and fiscal barriers to maternity leave and postpartum support during surgical training. JAMA Surg. Jul 1, 2021;156(7):647-653. [doi: ] [Medline: 34009280]

40. Castillo-Angeles M, Stucke RS, Rosenkranz KM, Smink DS, Rangel EL. Paternity Leave During Surgical Training: Perspectives of Male Residents. J Surg Educ. Nov 2022;79(6):e85-e91. [doi: ]

108. Castillo-Angeles M, Smink DS, Rangel EL. Perspectives of general surgery program directors on paternity leave during surgical training. JAMA Surg. Feb 1, 2022;157(2):105-111. [doi: 10.1001/jamasurg.2021.6223] [Medline: 34851404]

28. Corbisiero MF, Acker SN, Bothwell S, Christian N. Transforming perceptions: the impact of a formal parental leave policy on surgical trainees. J Surg Educ. Jun 2024;81(6):816-822. [doi: ] [Medline: 38677898]

124. Engelbrecht-Wiggans EA, Sundel MH, Newland JJ, Seyoum N, Brown RF. Parental leave policies in general surgery residencies. Am J Surg. Jul 2024;233:25-28. [doi: ] [Medline: 38160066]

48. Freudenberger DC, Riner AN, Herremans KM, et al. A gravid situation: general surgery faculty support for pregnant surgical residents. J Surg Res. Jul 2024;299:9-16. [doi: ] [Medline: 38677003]

60. Janczewski LM, Buchheit JT, Golisch KB, et al. Contemporary evaluation of work-life integration and well-being in US surgical residents: a national mixed-methods study. J Am Coll Surg. Dec 1, 2024;239(6):515-526. [doi: ] [Medline: 38920301]

61. Kling SM, Slashinski MJ, Green RL, Taylor GA, Dunham P, Kuo LE. Parental leave experiences for the non-childbearing general surgery resident parent: a qualitative analysis. Surgery. Nov 2024;176(5):1320-1326. [doi: ] [Medline: 38910045]

59. Li RD, Janczewski LM, Eng JS, et al. Pregnancy and parenthood among US surgical residents. JAMA Surg. Oct 1, 2024;159(10):1127-1137. [doi: ] [Medline: 39018050]

79. Ouyang K, Huang IA, Wagner JP, et al. Persistence of gender bias over four decades of surgical training. J Surg Educ. 2021;78(6):1868-1877. [doi: ] [Medline: 34294569]

82. Rangel EL, Lyu H, Haider AH, Castillo-Angeles M, Doherty GM, Smink DS. Factors associated with residency and career dissatisfaction in childbearing surgical residents. JAMA Surg. Nov 1, 2018;153(11):1004-1011. [doi: ] [Medline: 30073246]

11. Rangel EL, Smink DS, Castillo-Angeles M, et al. Pregnancy and motherhood during surgical training. JAMA Surg. Jul 1, 2018;153(7):644-652. [doi: ] [Medline: 29562068]

83. Rangel EL, Castillo-Angeles M, Marincola Smith P, Terhune KP. Parental support policies and benefits: a surgical training program report card of transparency. Ann Surg. Jun 1, 2023;277(6):938-943. [doi: ] [Medline: 35837953]

88. Sandler BJ, Tackett JJ, Longo WE, Yoo PS. Pregnancy and parenthood among surgery residents: results of the first nationwide survey of general surgery residency program directors. J Am Coll Surg. Jun 2016;222(6):1090-1096. [doi: ] [Medline: 26776357]

91. Shifflette V, Hambright S, Amos JD, Dunn E, Allo M. The pregnant female surgical resident. Adv Med Educ Pract. 2018;9(365–9):365-369. [doi: ] [Medline: 29785149]

101. Zhang C, Garcia-Neuer M, Jogerst KM, Van Der Walt C, Chang Y, Nguyen MC. Reproductive planning and infertility: training the next generation of surgeons. Am J Surg. Dec 2024;238:115886. [doi: ] [Medline: 39128441]

102. Zmijewski P, Aleman C, Panzica N, et al. Does your gender impact resident operative experience? a multi- institutional qualitative study. J Surg Educ. Feb 2025;82(2):103368. [doi: ] [Medline: 39709934]

92. Szender JB, Grzankowski KS, Eng KH, Odunsi K, Frederick PJ. Evaluation of satisfaction with work-life balance among U.S. gynecologic oncology fellows: a cross-sectional study. Gynecol Oncol Rep. Apr 2016;16:17-20. [doi: ] [Medline: 27331129]

51. Gaffley M, Hernandez S, Riera KM, Anzola S. Survey on the perceptions of pregnancy and parenthood in trainees: advances, obstacles, and growth opportunities. J Surg Res. Mar 2024;295:477-486. [doi: ] [Medline: 38070262]

58. Huynh M, Wang A, Ho J, Herndon CN, Aghajanova L. Fertility preservation and infertility treatment in medical training: an assessment of residency and fellowship program directors’ attitudes. Womens Health Rep (New Rochelle). 2021;2(1):576-585. [doi: ] [Medline: 35141706]

126. Magudia K, Ng TSC, Bick AG, et al. Parenting while in training: a comprehensive needs assessment of residents and fellows. J Grad Med Educ. Apr 2020;12(2):162-167. [doi: ] [Medline: 32322349]

73. Mundschenk MB, Krauss EM, Poppler LH, et al. Resident perceptions on pregnancy during training: 2008 to 2015. Am J Surg. Oct 2016;212(4):649-659. [doi: ] [Medline: 27575602]

84. Rangel EL, Castillo-Angeles M, Changala M, Haider AH, Doherty GM, Smink DS. Perspectives of pregnancy and motherhood among general surgery residents: A qualitative analysis. Am J Surg. Oct 2018;216(4):754-759. [doi: ] [Medline: 30072028]

106. Tatarian T, Anderson B, Pucci MJ, et al. Impact of surgeon workload on pregnancy outcomes in surgical trainees and faculty. Ann Surg. Jan 1, 2026;283(1):108-114. [doi: ] [Medline: 38939968]

96. Warner L, Hunter Guevara L, Watson A, Farmer S, Mehta R, Homme J. Changes in attitudes, beliefs, and experiences related to pregnancy during graduate medical education training from 2005 to 2021. Biomol Biomed. May 2, 2024;24(3):606-611. [doi: ] [Medline: 38149830]

80. Parker SL, Conner CR, Mata DA, et al. Factors associated with pregnancy and perinatal outcomes in female neurosurgeons: a cross-sectional study. Neurosurg. 2021;88(4):884-889. [doi: ]

89. Sanusi O, Kalu RU, Obayashi J, et al. Parental leave in neurosurgery: a US cross-sectional study. Neurosurg Pract. 2024;5(4). [doi: ]

93. Thum JA, Chang D, Tata N, Liau LM. Neurosurgeons in 2020: the impact of gender on neurosurgical training, family planning, and workplace culture. Neurosurg Focus. Mar 1, 2021;50(3):E11. [doi: ]

47. Esfandiari N, Litzky J, Sayler J, Zagadailov P, George K, DeMars L. Egg freezing for fertility preservation and family planning: a nationwide survey of US obstetrics and gynecology residents. Reprod Biol Endocrinol. Jan 29, 2019;17(1):16. [doi: ] [Medline: 30696433]

54. Hariton E, Matthews B, Burns A, Akileswaran C, Berkowitz LR. Pregnancy and parental leave among obstetrics and gynecology residents: results of a nationwide survey of program directors. Am J Obstet Gynecol. Aug 2018;219(2):199. [doi: ] [Medline: 29673570]

105. Martin CE, Hipp HS, Kottke M, Haddad LB, Kawwass JF. Fertility, pregnancy, and postpartum: a survey of practicing Georgia obstetrician gynecologists. Matern Child Health J. Oct 2019;23(10):1299-1307. [doi: ] [Medline: 31236824]

69. Mattei LH, Polan RM. Contemporary parental leave policies and practice among obstetrics and gynecology trainees and faculty. Clin Obstet Gynecol. Sep 1, 2024;67(3):524-530. [doi: ] [Medline: 38832715]

56. Huh DD, Wang J, Fliotsos MJ, et al. Association between parental leave and ophthalmology resident physician performance. JAMA Ophthalmol. Nov 1, 2022;140(11):1066-1075. [doi: ] [Medline: 36173610]

85. Reilly G, Tipton C, Liberman P, Berkenstock M. Attitudes toward parental leave and breastfeeding during ophthalmology residency. Can J Ophthalmol. Jun 2022;57(3):175-187. [doi: ] [Medline: 33789088]

118. Tao BKL, Hehar HK, Tien CW, Shunmugam M, Noureddin G, Khosa F. Gender of faculty leadership and online advertised parental leave policies for American ophthalmology residency programs. JFO Open Ophthalmology. Jun 2024;6:100106. [doi: ]

97. Wang KM, Lee B, Woreta FA, et al. Parental leave policy for ophthalmology residents: results of a nationwide cross-sectional study of program directors. J Surg Educ. May 2021;78(3):785-794. [doi: ]

127. Mercurio AM, Lynch OL, Shubin Stein BE, et al. Very few ACGME-accredited orthopaedic surgery residency programs have web-accessible leave policies dedicated to parental leave for residents, despite ACGME requirements. Clin Orthop Relat Res. Oct 1, 2024;482(10):1779-1785. [doi: ] [Medline: 38662935]

74. Mulcahey MK, Nemeth C, Trojan JD, OʼConnor MI. The perception of pregnancy and parenthood among female orthopaedic surgery residents. J Am Acad Orthop Surg. Jul 15, 2019;27(14):527-532. [doi: ] [Medline: 30499893]

76. Nguyen CV, Luong M, Weiss JM, Hardesty C, Karamitopoulos M, Poon S. The cost of maternity leave for the orthopaedic surgeon. J Am Acad Orthop Surg. Nov 15, 2020;28(22):e1001-e1005. [doi: ] [Medline: 32079849]

86. Reid DBC, Shah KN, Lama CJ, Kosinski LR, Daniels AH, Eberson CP. Parenthood among orthopedic surgery residents: assessment of resident and program director perceptions on training. Orthopedics. 2021;44(2):98-104. [doi: ] [Medline: 33561867]

87. Ruse S, Bergman R, Crawford E. Pregnancy in orthopaedic residents: peripartum barriers identified. JB JS Open Access. 2022;7(4):e22.00098. [doi: ] [Medline: 36601291]

128. Siljander BR, Van Nortwick SS, Flakne JC, Van Heest AE, Bohn DC. What proportion of orthopaedic surgery residency programs have accessible parental leave policies, and how generous are they? Clin Orthop Relat Res. 2020;478(7):1506-1511. [doi: ]

122. Weiss J, Teuscher D. What provisions do orthopaedic programs make for maternity, paternity, and adoption leave? Clin Orthop Relat Res. Sep 2016;474(9):1945-1949. [doi: ] [Medline: 27075331]

44. Diaz D, Freburg-Hoffmeister DL, Austin TM, Nyshadham S, Abramowicz S. Parental policy in oral and maxillofacial surgery residency programs is necessary but not available: a cross-sectional survey of oral and maxillofacial surgery residents’ attitudes toward parental leave. J Oral Maxillofac Surg. Dec 2021;79(12):2404-2410. [doi: ] [Medline: 34547262]

112. Tang AL, Miller A, Hauff S, et al. Maternity and paternity leave in otolaryngology residency training in the United States. Laryngoscope. May 2019;129(5):1093-1099. [doi: ] [Medline: 30315567]

36. Bourne DA, Chen W, Schilling BK, Littleton EB, Washington KM, De La Cruz C. The impact of plastic surgery training on family planning and prenatal health. Plast Reconstr Surg. Nov 2019;144(5):1227-1236. [doi: ] [Medline: 31688771]

49. Garza RM, Weston JS, Furnas HJ. Pregnancy and the plastic surgery resident. Plast Reconstr Surg. Jan 2017;139(1):245-252. [doi: ] [Medline: 28027263]

113. Wallace CC, Edmunds RW, Bourne D, Wong L. Parenting in plastic surgery residency. Plast Reconstr Surg. 2022;149(6):1465-1469. [doi: ]

103. Acker SN, Corbisiero MF, Romano J, et al. Attitudes of surgical trainees and faculty towards parental leave during surgical training. J Surg Educ. Sep 2024;81(9):1239-1248. [doi: ] [Medline: 38971678]

32. Altieri MS, Salles A, Bevilacqua LA, et al. Perceptions of surgery residents about parental leave during training. JAMA Surg. Oct 1, 2019;154(10):952-958. [doi: ] [Medline: 31389989]

34. Bernal IC, Moon SL, Hotta M, Newman MI. Residents’ perspectives of pregnancy and growing a family during surgical training: a review of the literature. Cureus. Apr 2024;16(4):e58335. [doi: ] [Medline: 38752085]

35. Bostock IC, Bayley EM, Antonoff MB. Barriers to parental leave during training: time for change. Am Surg. Mar 2023;89(3):452-456. [doi: ] [Medline: 34250836]

38. Castillo-Angeles M, Atkinson RB, Easter SR, et al. Pregnancy during surgical training: are residency programs truly supporting their trainees? J Surg Educ. 2022;79(6):e92-e102. [doi: ] [Medline: 35842402]

104. Mann H, Glazer T. Current state of safe pregnancy policies for the US surgical trainee. OTO Open. 2024;8(3):e172. [doi: ] [Medline: 39036338]

94. Todd AR, Cawthorn TR, Temple-Oberle C. Pregnancy and parenthood remain challenging during surgical residency: a systematic review. Acad Med. Oct 2020;95(10):1607-1615. [doi: ] [Medline: 32271231]

109. Kenyon LE, Malik R, Rodriguez D, Carmel ME. Urology program directors’ perception of pregnancy during residency. Urology. Jul 2021;153:75-80. [doi: ] [Medline: 33412219]

114. MacDonald SM, Raman JD. Widely variable parental leave practices for urology residency programs in the United States. Urology. Jul 2021;153:81-86. [doi: ]

68. Maloni K, Calligaro K, Reed A, Vani K, Dougherty M, Troutman D. Attitudes regarding pregnancy among vascular surgery trainees and program directors based on a survey of vascular residents, vascular fellows, and the Association of Program Directors in Vascular Surgery. Vasc Endovascular Surg. Aug 2022;56(6):561-565. [doi: ] [Medline: 35488400]

111. Morris L, Cronk NJ, Washington KT. Parenting during residency: providing support for Dr mom and Dr dad. Fam Med. Feb 2016;48(2):140-144. [Medline: 26950787]

72. Morris LE, Lindbloom E, Kruse RL, Washington KT, Cronk NJ, Paladine HL. Perceptions of parenting residents among family medicine residency directors. Fam Med. Nov 2018;50(10):756-762. [doi: ] [Medline: 30428104]

119. Wendling A, Paladine HL, Hustedde C, Kovar-Gough I, Tarn DM, Phillips JP. Parental leave policies and practices of US family medicine residency programs. Fam Med. Oct 4, 2019;51(9):742-749. [doi: ] [Medline: 31465110]

123. Stack SW, Finn KM, Kisielewski M, Law KL, Milne CK, Best JA. Parental leave policies in residency: a national survey of internal medicine program directors. Acad Med. Jul 1, 2022;97(7):1021-1028. [doi: ] [Medline: 35020617]

120. Ben-Zion S, Lehmann A, Price L, Burnett HQ, Michelson CD. The use of parenting electives in pediatric residency. Acad Pediatr. 2022;22(4):513-517. [doi: ] [Medline: 34864134]

29. Cree-Green M, Cree J, Urban K, Bunik M, Sass AE, Rosenberg A. A structured neonatal parenting elective: an approach for parenting leave during residency. Acad Pediatr. Jul 2020;20(5):595-599. [doi: ] [Medline: 32045680]

30. Creo AL, Anderson HN, Homme JH. Productive pumping: a pilot study to help postpartum residents increase clinical time. J Grad Med Educ. Apr 2018;10(2):223-225. [doi: ] [Medline: 29686765]

46. Dundon KMW, Powell WT, Wilder JL, et al. Parenthood and parental leave decisions in pediatric residency. Pediatrics. Oct 1, 2021;148(4). [doi: ]

81. Powell WT, Dundon KMW, Frintner MP, Kornfeind K, Haftel HM. Parenthood, parental benefits, and career goals among pediatric residents: 2008 and 2019. Pediatrics. Dec 1, 2021;148(6):57-64. [doi: ]

98. Wilder JL, Pingree EW, Hark CM, et al. Pediatric trainees as parents: perspectives on parenthood from pediatric resident parents. Acad Pediatr. Aug 2021;21(6):934-942. [doi: ] [Medline: 33878479]

99. Wilder JL, Hark CM, Marcus CH, et al. Pediatric trainees as parents: perspectives from a pandemic. Acad Pediatr. Aug 2021;21(6):927-933. [doi: ] [Medline: 33872822]

63. Kraus MB, Thomson HM, Dexter F, et al. Pregnancy and motherhood for trainees in anesthesiology: a survey of the American Society of Anesthesiologists. J Educ Perioper Med. 2021;23(1):E656. [doi: ] [Medline: 33778101]

62. Kraus MB, Malinzak EB, Chandrabose R, et al. A nationwide cross-sectional survey of anesthesiology fellowship program directors: attitudes on parental leave in residency and fellowship training. Womens Health Rep (New Rochelle). 2022;3(1):395-404. [doi: ] [Medline: 35652001]

107. McGough E, Martin TW, White P, Garvan C, Esfandiary L, Morey TE. Perceptions of anesthesiology residents concerning parental leave during residency. J Clin Anesth. Oct 2022;81:110910. [doi: ] [Medline: 35738027]

117. Morah O, Khosa F. Impact of department chair gender on paid parental leave across American anaesthesiology residencies. leader. Dec 2025;9(4):376-380. [doi: ]

90. Sharpe EE, Ku C, Malinzak EB, et al. A cross-sectional survey study of United States residency program directors’ perceptions of parental leave and pregnancy among anesthesiology trainees. Can J Anaesth. Oct 2021;68(10):1485-1496. [doi: ] [Medline: 34159567]

75. Mwakyanjala EJ, Cowart JB, Hayes SN, Blair JE, Maniaci MJ. Pregnancy and parenting during cardiology fellowship. J Am Heart Assoc. Jul 16, 2019;8(14):e012137. [doi: ] [Medline: 31286816]

77. Oliveros E, Burgess S, Nadella N, et al. Becoming a parent during cardiovascular training. J Am Coll Cardiol. May 31, 2022;79(21):2119-2126. [doi: ] [Medline: 35618349]

100. Yong CM, Abnousi F, Rzeszut AK, et al. Sex differences in the pursuit of interventional cardiology as a subspecialty among cardiovascular fellows-in-training. JACC Cardiovasc Interv. Feb 11, 2019;12(3):219-228. [doi: ] [Medline: 30660463]

53. Gracey LE, Cronin M, Shinkai K, Mathes EF. Program director and resident perspectives on new parent leave in dermatology residency. JAMA Dermatol. Oct 1, 2018;154(10):1222-1225. [doi: ] [Medline: 30167642]

57. Humphrey VS, Wyant WA, Brag KO. Family planning influences and perceptions among dermatology residents from ACGME-accredited training programs: a survey-based study. Arch Dermatol Res. May 11, 2024;316(5):159. [doi: ] [Medline: 38734865]

26. Chernoby KA, Pettit KE, Jansen JH, Welch JL. Flexible scheduling policy for pregnant and new parent residents: a descriptive pilot study. AEM Educ Train. Apr 2021;5(2):e10504. [doi: ] [Medline: 33898908]

110. MacVane CZ, Puissant M, Fix M, et al. Scheduling practices for pregnant emergency medicine residents. AEM Educ Train. Dec 2022;6(6):e10813. [doi: ] [Medline: 36425789]

31. Advani R, Saeed N, Arjonilla M, et al. Gender, marital, and parental barriers to pursuing gastroenterology fellowship: a multicenter analysis of a national survey and actionable solutions. Dig Dis Sci. Feb 2025;70(2):504-515. [doi: ] [Medline: 39762595]

42. David YN, Dixon RE, Kakked G, et al. Pregnancy and the working gastroenterologist: perceptions, realities, and systemic challenges. Gastroenterology. Sep 2021;161(3):756-760. [doi: ] [Medline: 34089733]

50. Gardiner CP, Desrochers L, Finn K, et al. Paying for parenthood: understanding parental leave policies in infectious disease fellowship. Open Forum Infect Dis. Feb 2024;11(2):ofad685. [doi: ] [Medline: 38390462]

45. Dixon A, Bansal N, Nicholas SB, Ostrow A, Kendrick J. A national survey of pregnancy and parenthood among nephrology trainees: a focus on nephrology fellowship. Clin J Am Soc Nephrol. Aug 1, 2024;19(8):984-994. [doi: ] [Medline: 38728092]

27. Conway SE, Vaswani PA, Budhu JA, et al. Development and impact of a progressive parental leave policy in a neurology residency. Neurology. Jun 7, 2022;98(23):973-979. [doi: ] [Medline: 35418451]

41. Conway SE, Wang W, Prasad S. Barriers to increasing paid parental leave in U.S. neurology residencies: a survey of program directors. BMC Med Educ. Apr 9, 2024;24(1):387. [doi: ] [Medline: 38594709]

67. Malapati SJ, Idossa D, Singh SRK, et al. Parent penalty: parental leave experiences of trainees and early-career faculty in oncology subspecialties. Chino F, editor. JCO Oncol Pract. Oct 2023;19(10):899-906. [doi: ] [Medline: 37708434]

70. McDeavitt JT, Appelbaum NP, Raddatz MM, Driscoll SW, Kinney CL. Taking leave during residency: types of absences and subsequent delays and variations in physical medicine and rehabilitation medical board pass rates. Am J Phys Med Rehabil. Jul 1, 2022;101(7 Suppl 1):S30-S34. [doi: ] [Medline: 35706116]

43. Dillinger RL. From requisite to right: assessing and addressing paid maternity leave in US psychiatry residency programs. Acad Psychiatry. Apr 2022;46(2):167-171. [doi: ] [Medline: 34559391]

64. Leandre FM, Sudak DM, Ginory A. Are psychiatry programs providing adequate parental leave to their residents? Acad Psychiatry. Apr 2022;46(2):162-166. [doi: ] [Medline: 34751936]

33. Baniel CC, Qu V, Ponce SB, et al. SUPPORT: SUrvey of Parental Leave POlicies of RadiaTion oncology programs and residency applicants. Adv Radiat Oncol. 2023;8(4):101207. [doi: ] [Medline: 37124316]

20. Beltrán Ponce S, Jagsi R, Florez N, et al. Can I leave? perspectives on parental leave and parenthood in medical training among program directors and trainees in oncologic specialties. J Womens Health. Feb 2024;33(2):218-227. [doi: ]

55. Holliday EB, Ahmed AA, Jagsi R, et al. Pregnancy and parenthood in radiation oncology, views and experiences survey (PROVES): results of a blinded prospective trainee parenting and career development assessment. Int J Radiat Oncol Biol Phys. Jul 2015;92(3):516-524. [doi: ]

78. Osborn VW, Doke K, Griffith KA, et al. A survey study of female radiation oncology residents’ experiences to inform change. Int J Radiat Oncol Biol Phys. Aug 1, 2019;104(5):999-1008. [doi: ] [Medline: 31108141]

52. Ghazi Sherbaf F, Lin DDM, Yousem DM. Parental leave policy in radiology residency programs: current status. J Am Coll Radiol. Sep 2020;17(9):1163-1171. [doi: ] [Medline: 32275902]

116. Hui DHF, Yakub M, Tiwana S, et al. Gender of department chair and paid parental leave benefits in academic radiology residency programs. Curr Probl Diagn Radiol. 2022;51(2):162-165. [doi: ] [Medline: 34949474]

121. Sherbaf FG, Lin DDM, Yousem DM. Parental leave and neuroradiology fellowships. AJNR Am J Neuroradiol. Aug 2020;41(8):1348-1354. [doi: ] [Medline: 32646942]

130. Wong TY, Huang JJ, Hoffmann JC, Flug JA, Cooke EA, Donnelly EF. Resident wellness in radiology as portrayed by departmental websites. Acad Radiol. Aug 2022;29(8):1259-1265. [doi: ] [Medline: 34400076]

125. Kasemodel K, Hemal K, Chen W, Wallace C, Bourne DA. Evolution in parental leave policies: plastic surgery and obstetrics/gynecology lead the way. Plast Reconstr Surg. Nov 1, 2022;150(5):1160-1168. [doi: ] [Medline: 36067473]

65. Lumpkin ST, Klein MK, Battarbee AN, Strassle PD, Scarlet S, Duke MC. Fellowship or family? a comparison of residency leave policies with the Family and Medical Leave Act. J Surg Res. Sep 2019;241:302-307. [doi: ] [Medline: 31048221]

66. Magudia K, Campbell SR, Rangel EL, et al. Medical specialty board parental, caregiver, and medical leave policy updates after 2021 American Board of Medical Specialties Mandate. JAMA. Nov 9, 2021;326(18):1867-1870. [doi: ] [Medline: 34751719]

71. Morgan JC, Owens T, Carmack MC, Braverman A, Class QA. Policy and perceptions of pregnancy during training among residents of various subspecialties. Postgrad Med J. Apr 22, 2025;101(1195):441-446. [doi: ] [Medline: 39574162]

95. Varda BK, Glover M 4th. Specialty board leave policies for resident physicians requesting parental leave. JAMA. Dec 11, 2018;320(22):2374-2377. [doi: ] [Medline: 30535211]
