# Supplementary material for: Parenthood in US Medical Training Across Specialty Groups: Scoping Review
Source: JMIR Med Educ. 2026 Jul 2;12:e87284. doi: 10.2196/87284 (PMC13325462; doi:10.2196/87284)
Supplement: Multimedia Appendix 3 [file mededu-v12-e87284-s003.docx]

| **Multimedia Appendix 3**. Sensitivity Analysis of Specialty‑Based Themes with Anesthesiology Reclassified as a Surgical Specialty | | | | | | | | | |
| --- | --- | --- | --- | --- | --- | --- | --- | --- | --- |
|  | **Total** (*N*=105), n (%) | | **Surgical** (*N*=64), n (%) | **PC** (*N*=11), n (%) | **MS** (N=25), n (%) | | **All** (*N*=5), n (%) | **Chi-square value(df), *P*** | **Fisher’s exact test** |
| **Individual** | 49 (46%) | | 33 (52%) | 3 (27%) | 13 (52%) | | 0 | 2.34(2), .31 | *P*=.34 |
| Knowledge/Belief | 20 (19%) | | 14 (22%) | 0 | 6 (24%) | | 0 | 3.14(2), .21 | *P*=.22 |
| Health | 26 (25%) | | 17 (27%) | 2 (18%) | 7 (28%) | | 0 | 0.41(2), .82 | *P*=.89 |
| Fertility | 18 (17%) | | 13 (20%) | 0 | 5 (20%) | | 0 | N/A | *P*=.31 |
| Financial constraints | 4 (4%) | | 3 (5%) | 1 (9%) | 0 | | 0 | N/A | *P*=.28 |
| **Interpersonal**  Positive n (%)  *Positive only excluded* | 68 (65%)  12 (11%)    62 (59%) | | 45 (70%)  5 (8%)  *40 (63%)* | 6 (55%)  3 (27%)  *3 (27%)* | 15 (60%)  3 (12%)  *12 (48%)* | | 2 (40%)  0  2 (40%) | 1.57 (2), .46    *5.37 (2), .07* | *P*=.44    *P*=.*07* |
| Colleague Burden  *Positive only excluded* | 45 (43%)  44 (42%) | | 30 (47%)  30 (47%) | 5 (45%)  *4 (36%)* | 8 (32%)    8 (32%) | | 2 (40%)  2 (40%) | 1.65 (2), .44  *1.43* (2), *.49* | *P*=.44  *P*=.*48* |
| **Bias/Stigma**  *Positive only excluded* | 37 (35%)  36 (34%) | | 27 (42%)  27 (42%) | 2 (18%)  *1 (9%)* | 6 (24%)  6 (24%) | | 2 (40%)  2 (40%) | 4.15 (2), .12  ***6.07 (2), .05**** | *P*=.15  ***P*=.*05****** |
| **Faculty Support**  *Positive only excluded* | 37 (35%)  32 (30%) | | 25 (39%)  25 (39%) | 4 (36%)  *1 (9%)* | 6 (24%)  *5 (17%)* | | 2 (40%)  2 (40%) | 1.8 (2), .41  ***5.83*** (2), ***.05**** | *P*=.42  *P*=*.07* |
| **Trainee Support**  *Positive only excluded* | 39 (37%)  34 (32%) | | 27 (42%)  27 (42%) | 4 (36%)  *2 (18%)* | 6 (24%)  *5 (17%)* | | 2 (40%)  2 (40%) | 2.55 (2), .28  *5.32 (2), .07* | *P*=.28  *P*=.*08* |
| Perceived performance  *Positive only excluded* | 28 (27%)   25 (24%) | | 20 (31%)  20 (31%) | 2 (18%)  *1 (9%)* | 5 (20%**)**  *4 (16%)* | | 1 (20%)  1 (20%) | 1.64 (*2),* .44  3.9 (*2),* .14 | *P*=.55  *P*=.17 |
| **Patient relationships**  *Positive only excluded* | 7 (7%)  6 (6%) | | 3 (5%)  3 (5%) | 4 (36%)  *3 (27%)* | 0  0 | | 0  0 | N/A | ***P*=.003***  ***P*=.*01**** |
| Family relationships  *Positive only excluded* | 10 (10%)  9 (9%) | | 6 (9%)  6 (9%) | 3 (27%)  *2 (18%)* | 1 (4%)  1 (4%) | | 0  0 | N/A | *P*=.*11*  *P*=.37 |
| Role Model/Mentors  *Positive only excluded* | 10 (10%)  9 (9%) | | 8 (13%)  *7 (11*%) | 2 (18%)   2 (18%) | 0  0 | | 0  0 | N/A | *P*=.*08*  *P*=.15 |
| Joy of parenthood | 2 (2%) | | 0 | *2 (18%)* | 0 | | 0 | N/A | ***P*=.*01**** |
| **Organization/Community**  Positive n (%)  *Positive only excluded* | 87 (83%)  20 (19%)  73 (70%) | | 53 (83%)  5 (8%)  *48 (75%)* | 11 (100%)  8 (73%)  *5 (45%)* | 21 (84%)  7 (28%)  *15 (60%)* | | 2 (40%)  0  2 (40%) | *4.75 (2), .09* | *P*=.44    *P*=.08 |
| Childcare | 26 (25%) | | 17 (27%) | 4 (36%) | 5 (20%) | | 0 | 1.09 (*2),* .58 | *P*=.54 |
| **Culture of support** for pregnancy, parenthood  *Positive only excluded* | 40 (38%)    38 (36%) | | 29 (45%)    29 (45%) | 4 (36%)    *2 (18%)* | 5 (20%)    5 (20%) | | 2 (40%)    2 (40%) | 4.9 (*2),* .09    ***6.7 (2), .04**** | *P*=.08    ***P*=.*04**** |
| Culture of support for PL  *Positive only excluded* | 55 (52%)  46 (44%) | | 32 (50%)  32 (50%) | 7 (64%)  *3 (27%)* | 15 (60%)  *13 (52%)* | | 1 (20%)  1 (20%) | 1.2 (*2),* .55  *2.16 (2), .34* | *P*=.59  *P*=.*37* |
| Financial barriers | 7 (7%) | | 5 (8%) | 0 | 2 (8%) | | 0 | N/A | *P*>.99 |
| PL coverage system | 37 (35%) | | 19 (30%) | 5 (45%) | 13 (52%) | | 0 | 4.22 (*2),* .12 | *P*=*.12* |
| Lactation space  *Positive only excluded* | 22 (21%)  19 (18%) | | 12 (19%)  12 (19%) | 5 (45%)  *2 (18%)* | 5 (20%)  5 (20%) | | 0  0 | 3.98 (*2),* .14 | *P*=*.17*  *P>.99* |
| Lactation time | 18 (17%) | | 10 (16%) | 3 (27%) | 5 (20%) | | 0 | N/A | *P*=.53 |
| Schedule Rigor/Flexibility  *Positive only excluded* | 42 (40%)  34 (32%) | | 27 (42%)  *26 (41%)* | 4 (36%)  *1 (9%)* | 10 (40%)  *6 (24%)* | | 1 (20%)  1 (20%) | 0.15 (*2),* .93  *5.44 (2), .07* | *P*>.99  *P*=.07 |
| PD knowledge | 10 (10%) | | 5 (8%) | 1 (9%) | 4 (16%) | | 0 | N/A | *P*=.47 |
| Gender of chair or PD | 10 (10%) | | 6 (9%) | 2 (18%) | 2 (8%) | | 0 | N/A | *P*=.61 |
| **Policy**  Positive (study n)  *Positive only excluded* | 83 (79%)  5 (5%)  79 (75) | | 51 (80%)  2 (3%)  *49 (77%)* | 6 (55%)  2 (18%)  *4 (36%)* | 21 (84%)  1 (3%)  *20 (80%)* | | 5 (100%)  5 (100%) | 4.16 (*2),* .12    ***8.52 (2), .02**** | *P*=.17    ***P*=.*02**** |
| PL Policy Existence  *Positive only excluded* | 61 (58%)  58 (55%) | | 36 (56%)  *34 (53%)* | 4 (36%)  *3 (27%)* | 18 (72%)  *17 (68%)* | | 3 (60%)  3 (60%) | 4.21 (*2),* .12  *5.16 (2), .08* | *P*=.14  *P*=.08 |
| PL length  *Positive only excluded* | 52 (50%)  50 (48%) | | 31 (48%)  31 (48%) | 4 (36%)  *3 (27%)* | 13 (52%)  *12 (48%)* | | 4 (80%)  4 (80%) | 0.76 (*2),*.68  *1.75 (2), .41* | *P*=*.*72  *P*=*.45* |
| Childbearing PL | 52 (50%) | | 34 (53%) | 5 (45%) | 12 (48%) | | 1 (20%) | 0.34 (*2),* .84 | *P*=.87 |
| Nonchildbearing PL | 37 (35%) | | 24 (38%) | 3 (27%) | 9 (36%) | | 1 (20%) | 0.43 (*2),* .81 | *P*=.86 |
| Adoptive/Nontraditional PL | 5 (5%) | | 2 (3%) | 0 | 3 (12%) | | 0 | N/A | *P*=.19 |
| Paid PL | 38 (36%) | | 19 (30%) | 6 (55%) | 11 (44%) | | 2 (40%) | 3.44 (*2),* .18 | *P*=.19 |
| Underuse of PL | 17 (16%) | | 8 (13%) | 3 (27%) | 6 (24%) | | 0 | N/A | *P*=.17 |
| Lactation Policy | 9 (9%) | | 4 (6%) | 0 | 4 (16%) | | 1 (20%) | N/A | *P*=.26 |
| Board Policy | 20 (19%) | | 11 (17%) | 1 (9%) | 5 (20%) | | 3 (60%) | N/A | *P*=.79 |
| Institutional Policy | 25 (24%) | | 14 (22%) | 1 (9%) | 9 (36%) | | 1 (20%) | 3.47 (*2),* .17 | *P*=.20 |
| Consistency of policies | 13 (12%) | | 9 (14%) | 1 (9%) | 3 (10%) | | 0 | N/A | *P*>.99 |
| **Interactions**  Positive (study n)  *Positive only excluded* | 78 (74%)  13 (12%)  71 (68%) | | 48 (75%)  5 (8%)  *43 (67%)* | 7 (64%)  4 (36%)  *6 (55%)* | 20 (80%)  3 (12%)  *17 (68%)* | | 3 (60%)  1 (10%)   2 (40%) | 1.09 (*2),* .58    *0.73 (2), .69* | *P*=.61    *P*=.*70* |
| Training Extension | 37 (35%) | | 21 (33%) | 4 (36%) | 9 (36%) | | 3 (60%) | 0.11 (*2),* .95 | *P*=.95 |
| Parenthood Postponement | 28 (27%) | | 18 (28%) | 1 (9%) | 8 (32%) | | 1 (20%) | 2.15 (*2),* .34 | *P*=.41 |
| Training & Parenthood incompatibility | 12 (11%) | | 11 (17%) | 1 (9%) | 0 | | 0 | N/A | *P*=.06 |
| Training experience  *Positive only excluded* | 29 (28%)  27 (26%) | | 18 (28%)  *16 (25%)* | 4 (36%)  4 (36%) | 7 (28%)  7 (28%) | | 0  0 | 0.33 (*2),* .84  0.63 (*2),* .73 | *P*=.84  *P*=.66 |
| Specialty Choice | 16 (15%) | | 10 (16%) | 1 (9%) | 4 (16%) | | 1 (20%) | N/A | *P*=.99 |
| Career  *Positive only excluded* | 28 (27%)  21 (20%) | | 16 (25%)  *12 (19%)* | 3 (27%)  *2 (18%)* | 9 (36%)  *7 (28%)* | | 0  0 | 1.08 (*2),* .58  *0.99 (2), .61* | *P*=.56  *P*=.57 |
| Breastfeeding Success  *Positive only excluded* | 14 (13%)  13 (12%) | | 6 (9%)  6 (9%) | 3 (27%)  *2 (18%)* | 5 (20%)  5 (20%) | | 0  0 | N/A | *P*=.12  *P*=.26 |
| Combination and wellbeing/stress | 18 (17%)   17 (16%) | | 13 (20%)  13 (20%) | 3 (27%)  3 (27%) | 2 (8%)  *1 (4%)* | | 0  0 | N/A   N/A | *P*=.*28*  *P*=.09 |
| **Table Legend** | | n: number  MS: medical subspecialty  PC: primary care | | | | PD: program director  PL: parental leave  *: statistically significant at p<0.05 | | | |
